# Functional characterization of hydroxyproline-*O*-galactosyltransferases for Arabidopsis arabinogalactan-protein synthesis

**DOI:** 10.1186/s12870-021-03362-2

**Published:** 2021-12-13

**Authors:** Dasmeet Kaur, Michael A. Held, Mountain R. Smith, Allan M. Showalter

**Affiliations:** 1 Molecular and Cellular Biology Program, Ohio University, Athens, OH 45701-2979 USA; 2 Department of Environmental & Plant Biology, Ohio University, Athens, OH 45701-2979 USA; 3 Department of Chemistry & Biochemistry, Ohio University, Athens, OH 45701-2979 USA

**Keywords:** Arabidopsis, Arabinogalactan-proteins, Hydroxyproline-galactosyltransferase, Reproduction, Pollen, β-Yariv, Root length, Seed set, Seed germination

## Abstract

**Background:**

Arabinogalactan-proteins (AGPs) are structurally complex hydroxyproline-rich cell wall glycoproteins ubiquitous in the plant kingdom. AGPs biosynthesis involves a series of post-translational modifications including the addition of type II arabinogalactans to non-contiguous Hyp residues. To date, eight Hyp-galactosyltransferases (Hyp-GALTs; GALT2-GALT9) belonging to CAZy GT31, are known to catalyze the addition of the first galactose residues to AGP protein backbones and enable subsequent AGP glycosylation. The extent of genetic redundancy, however, remains to be elucidated for the *Hyp-GALT* gene family.

**Results:**

To examine their gene redundancy and functions, we generated various multiple gene knock-outs, including a triple mutant (*galt5 galt8 galt9*), two quadruple mutants (*galt2 galt5 galt7 galt8, galt2 galt5 galt7 galt9*), and one quintuple mutant (*galt2 galt5 galt7 galt8 galt9*), and comprehensively examined their biochemical and physiological phenotypes. The key findings include: AGP precipitations with β-Yariv reagent showed that *GALT2, GALT5, GALT7, GALT8* and *GALT9* act redundantly with respect to AGP glycosylation in cauline and rosette leaves, while the activity of *GALT7, GALT8* and *GALT9* dominate in the stem, silique and flowers. Monosaccharide composition analysis showed that galactose was decreased in the silique and root AGPs of the *Hyp-GALT* mutants. TEM analysis of *25789* quintuple mutant stems indicated cell wall defects coincident with the observed developmental and growth impairment in these *Hyp-GALT* mutants. Correlated with expression patterns, *galt2*, *galt5*, *galt7*, *galt8,* and *galt9* display equal additive effects on insensitivity to β-Yariv-induced growth inhibition, silique length, plant height, and pollen viability. Interestingly, *galt7*, *galt8,* and *galt9* contributed more to primary root growth and root tip swelling under salt stress, whereas *galt2* and *galt5* played more important roles in seed morphology, germination defects and seed set. Pollen defects likely contributed to the reduced seed set in these mutants.

**Conclusion:**

Additive and pleiotropic effects of *GALT2*, *GALT5*, *GALT7*, *GALT8* and *GALT9* on vegetative and reproductive growth phenotypes were teased apart via generation of different combinations of *Hyp-GALT* knock-out mutants. Taken together, the generation of higher order *Hyp-GALT* mutants demonstrate the functional importance of AG polysaccharides decorating the AGPs with respect to various aspects of plant growth and development.

**Supplementary Information:**

The online version contains supplementary material available at 10.1186/s12870-021-03362-2.

## Background

Arabinogalactan-proteins (AGPs) are a complex family of extracellular, hydroxyproline (Hyp)-rich glycoproteins (HRGPs) found throughout the plant kingdom, from aquatic brown and green algae (e.g. *Ectocarpus siliculosus*) [1, 2] to terrestrial angiosperms [3, 4]. Their complexity is reflected in the multiple AGP subfamilies/core proteins and in the extensive post-translational modifications of these core proteins, which includes hydroxylation of proline residues to form hydroxyproline (Hyp), removal of signal peptide sequences which facilitate extracellular secretion, addition of glycosylphosphatidylinositol (GPI) lipid anchors to allow for attachment of many AGPs on the outer leaflet of the plasma membrane [4–6], and the addition of type II arabinogalactan (AG) polysaccharide side chains catalyzed by diverse set of glycosyltransferases (GTs). AGPs are found at the plasma membrane-cell wall interface, in the cell wall and in plant exudates of most cells, tissues, and organs [7].

Although AGPs account for less than 10% of the plant cell wall matrix, they are implicated to function in a number of important plant growth and development processes including cell expansion, cell division, programmed cell death, somatic embryogenesis, root formation and development, xylem differentiation, responses to abiotic stress, and hormone signaling [8–13]. AGPs also play important roles in the food, pharmaceutical and mining industries given their remarkable emulsification, adhesion, and lubrication properties. Using bioinformatic analysis, Showalter et al. (2010) identified 85 AGP genes in *Arabidopsis* and classified them into various subfamilies including classical AGPs, lysine-rich AGPs, AG peptides, fasciclin-like AGPs, and several other chimeric AGPs. Despite the immense importance of AGPs to plants as well as humans, their molecular mechanisms of action with respect to their functions remains elusive.

Given that AGPs generally consist of 1–10% (w/w) protein and ~ 90–98% (w/w) carbohydrate, it is likely that the type II AG sugars of AGPs constitute their functionally interactive surface and are integral to their mechanisms of action [14, 15]. Typically, type II AGs consist of β-1,3-linked galactan main chains substituted with variable lengths of β-1,6-galactan side chains (up to 100 to 150 sugar residues) which contain L-arabinose along with other sugar residues [16–18].

To date, 20 GTs involved in the synthesis of type II AGs have been identified and characterized [19], including two β-1,3-galactosyltransferases, namely *At1g77810* and *At1g33430* (KNS4)*,* in the GT31 family (http://www.cazy.org/GT31_all.html) [20]*,* two β-1,6-galactosyltransferases GALT31A and GALT29A in GT31 family [21, 22], three β-glucuronosyltransferases, GlcAT14A, GlcAT14B, and GlcAT14C, from GT14 family [23, 24], two α-fucosyl-transferases FUT4 and FUT6, in GT37 [25–27] and one β-arabinosyl-transferase RAY1 from the GT77 family [28].

In addition to the recently identified *Hyp-galactosyltransferase* (*Hyp-GALT) At1g22015* [29], eight *Hyp-GALT* genes have been identified and characterized so far. These eight genes reside in two separate clades within the GT31 family in the CAZy database. Of all the GTs involved in the glycosylation of AGPs, the *Hyp-GALTs* are the most critical as they add the first galactose onto the Hyp residues of the protein backbone and generate the acceptor for subsequent glycosylation events. Five *Hyp-GALTs,* designated as *GALT2–6* by the Showalter lab, were identified by bioinformatic analysis and verified by heterologous expression in *Pichia pastoris* and *Nicotiana benthamiana* using an in vitro enzyme assay [30, 31]. By contrast, the other three *Hyp-GALTs*, designated as *HPGT1–3*, were identified via affinity chromatography with an AGP peptide followed by protein sequencing and heterologous expression coupled with an in vitro enzyme assay [32]. For simplicity, these eight *Hyp-GALTs* will be referred to as *GALT2–9*, such that *HPGT1–3* are renamed as *GALT7–9*. One *Hyp-GALT* clade in GT31 consists of *GALT2* (*At4g21060*), *GALT3* (*At3g06440*), *GALT4* (*At1g27120*), *GALT5* (*At1g74800*), and *GALT6* (*At5g62620*), and these genes encode proteins having a GALT domain (pfam01762) as well as a GALECTIN (pfam00337) domain. A second *Hyp-GALT* clade in GT31 consists of *GALT7/HPGT1* (*At5g53340*), *GALT8/HPGT2* (*At4g32120*) and *GALT9/HPGT3* (*At2g25300*), and these genes encode proteins that only have a GALT domain and lack a GALECTIN domain [19, 32, 33].

Single genetic knock-out mutants for these eight *Hyp-GALTs* demonstrated subtle or no discernable plant phenotypes compared to wild-type (WT) plants. The *galt2 galt5* double mutant, however, displayed pleiotropic effects including impaired root growth, root tip swelling in response to salt stress and a reduction in seed coat mucilage [34]. Whereas the *hpgt1 hpgt2 hpgt3* triple mutant exhibited reductions in plant height, leaf size, seed set, silique length as well as an increase in root length. Moreover, Zhang et al. (2021) reported gene redundancy of a GT31 galactosyl-transferases belonging to a single clade (with both GALT and GALECTIN domains) by generating a triple mutant *galt3 galt4 galt6* and quintuple mutant *galt2 galt3 galt4 galt5 galt6* using CRISPR/Cas9 gene editing/multiplexing approach, which exhibited reduced overall growth, impaired root growth, abnormal pollen, and reduced seed set with shorter siliques [35].

Given the complexity in understanding the roles of multiple genes belonging to two subfamilies (clades), we hypothesized that knocking out multiple *Hyp-GALTs* in different combinations will allow us to examine the extent of gene redundancy in two *Hyp-GALT* clades with structural domain differences and to elucidate the biological contributions of these two different clades on vegetative and reproductive organs by extensive phenotypic analysis. Here, we report the generation and extensive characterization of multiple *Hyp-GALT* (T-DNA) gene knockout mutants, including a triple mutant (*galt5 galt8 galt9*), two quadruple mutants (*galt2 galt5 galt7 galt8, galt2 galt5 galt7 galt9*) and one quintuple mutant (*galt2 galt5 galt7 galt8 galt9*) and discuss the new insights with respect to *Hyp-GALT* partial genetic redundancy, AGP biosynthesis and AGP functions.

## Results

### Expression profiles of eight *Hyp-GALTs* in the GT31 family

Expression of the eight *Hyp-GALTs* genes was examined in various vegetative and floral organs/tissues using publicly available RNA-seq data sets with Araport (Supplemental Fig. 1). This in silico analysis indicated mostly overlapping tissue expression patterns for the eight genes throughout the plant except pollen which showed unique expression pattern, corroborating previous studies based on microarray and quantitative RT-PCR data (Basu et al., 2015; Ogawa-Ohnishi and Matsubayashi, 2015; Basu et al., 2016). All the *Hyp-GALT*s genes were expressed in multiple plant organs/tissues including stem/aerial, carpel, inflorescence, leaf, pollen, root, seedling, root and shoot apical meristem. Among the eight genes, *GALT7* generally displayed the highest expression levels, particularly in inflorescence and root tissues.

In addition, transcriptomic analysis data of laser-capture micro-dissected seeds as depicted in the BAR eFP browser [36] showed high but distinct expression patterns for the *Hyp-GALT* genes during various stages of seed development (Supplemental Fig. 2). *GALT2*, *GALT3*, *GALT5* and *GALT8* were highly expressed in the seed coat at the pre-globular and globular stages of seed development, while *GALT4*, *GALT7*, *GALT8* and *GALT9* were highly expressed in the micropylar endosperm. These expression patterns during seed development pointed towards a possibility of germination and seed coat defects in *Hyp-GALT* mutants.

### Generation of higher-order *Hyp-GALT* knock out mutants

**Fig. 1:**
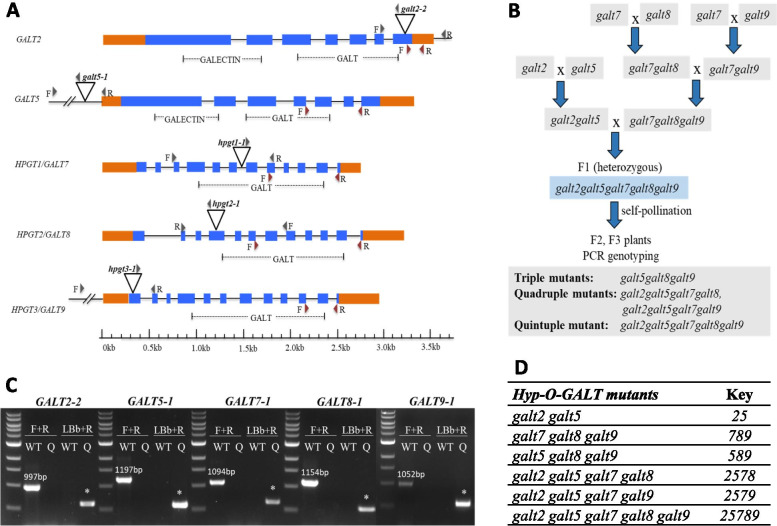
Single T-DNA insertional mutant information, the strategy for generating higher-order *Hyp-GALT* mutants and the list of mutants obtained after screening. **A** Schematic gene models for *GALT2*, *GALT5*, *GALT7*, *GALT8* and *GALT9*, including the locations of T-DNA mutant insertions and primers used for PCR and qRT-PCR. The exon/intron structures are indicated; introns are drawn as lines and exons are drawn as rectangles, with blue rectangles representing coding sequences and orange rectangles representing UTRs. Sites of T-DNA insertions (marked as triangles) and locations of PCR primer sequences (grey arrowheads above the genes) and qRT-PCR primer sequences (red arrowheads below the genes) used for PCR screening and qRT-PCR, respectively, are indicated. The grey arrowheads above the T-DNA insertion indicate the location of the LBb1.3 T-DNA primer. The predicted GALECTIN (Pfam 00337) and GALT (Pfam 01762) domains are denoted by dashed lines. Information on *Hyp-GALTs* genes and their protein lengths ranging from 338 to 741 amino acids are provided in Supplementary Table 1. **B** The crossing strategy for generating a set of higher-order *Hyp-GALT* gene mutants. **C** Confirmation of the genotype of the *galt2 galt5 galt7 galt8 galt9* quintuple mutant (marked as Q) by PCR. For example, *galt2–2* genotyping produced a single band of 449–779 bp in size from the homozygous *galt2* allele (*), whereas the WT allele (WT) produced a single band of 997 bp in size. Genotyping primer information and the sizes of the expected amplicon of each mutant allele are provided in Supplementary Table S2. **D** List of higher-order *Hyp-GALT* mutants and the key used in this study for all subsequent figures

T-DNA insertion alleles GALT2, GALT5 [30] as well as GALT7, GALT8 and GALT9 [32] were used to generate multiple higher-order mutants (Fig. 1A and Supplemental Table 1). All the single alleles were reported to be T-DNA insertions in the introns, exons or UTRs: *galt2*–*2*, *galt8 (hpgt2–1)* and *galt9 (hpgt3–1)* had a T-DNA insertion in exon seven, four and one respectively, *galt5–1* had T-DNA insertion in 5’UTR whereas *galt7 (hpgt1–1)* had a T-DNA insertion in intron six. *galt2 galt5* double mutant and the *galt7 galt8 galt9 (hpgt1 hpgt2 hpgt3)* triple mutant, which were reported in previous studies [30, 32], were crossed to generate a line heterozygous for all these *Hyp-GALT* genes, namely *galt2 galt5 galt7 galt8 galt9.* This line was self-pollinated and the resulting F2 and F3 plants were subjected to PCR genotyping to isolate various triple, quadruple, and quintuple homozygous mutants (Fig. 1B). All five genes used in this study are located on different chromosomes or are far apart on the same chromosome in the *Arabidopsis* genome in one case. *GALT2, GALT5, GALT7, GALT8*, and *GALT9* are located on chromosome IV, I, V, IV and II, respectively. The *GALT2* and *GALT8* genes located 4.27 Mb apart on the long arm of the chromosome IV according to The National Center for Biotechnology Information [37].

The resulting mutants obtained from the F2 and F3 screening used in this study were *galt5 galt8 galt9*, *galt2 galt5 galt7 galt8*, *galt2 galt5 galt7 galt9* and *galt2 galt5 galt7 galt8 galt9* (see Key in Fig. 1D) along with WT (Col-0), *galt2 galt5* and *galt7 galt8 galt9* as controls. Figure 1C provides an example of genotype conformation for the quintuple mutant.

### Relative expression profiles of the *Hyp-GALT* genes in higher-order mutants

**Fig. 2:**
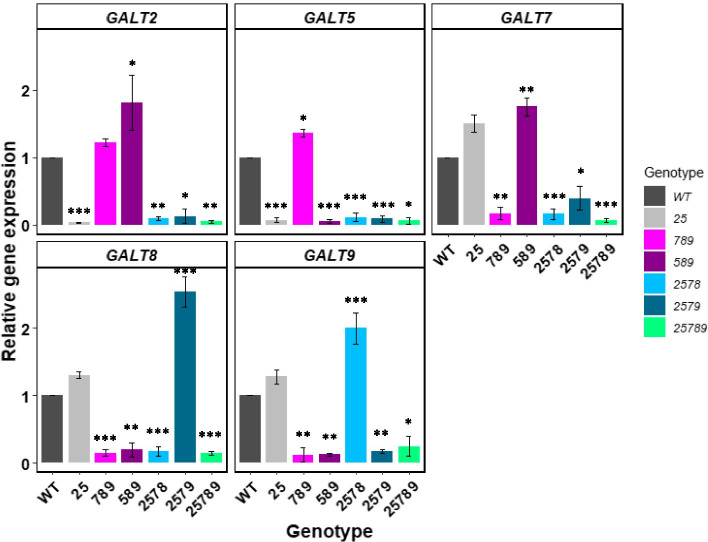
Relative expression profile of the five *Hyp-GALT* genes in higher-order *Hyp-GALT* mutants compared to WT determined via qRT-PCR. Total RNA was extracted from inflorescence samples collected from the various *Hyp-GALT* mutant plants at 40 days after germination (DAG). Expression levels were normalized to the *ACT2* gene (mean ± SE of three biological replicates). In addition, expression levels of each gene in WT plants were set to one. An analysis of variance (ANOVA) on these mutants yielded significant variation among conditions. A post hoc Tukey test was applied to see which groups were significantly different from wild-type (Col-0). *P < .05, **P < 0.005, ***P < 0.001

Quantitative reverse transcription (qRT-PCR) was performed to assess the expression of the five *Hyp-GALT* genes: *GALT2*, *GALT5*, *GALT7*, *GALT8* and *GALT9* genes from inflorescences collected from the plants grown on soil for 40 d (Fig. 2) which is in good agreement with qRT-PCR and RT-PCR of single allelic mutants and the *25* double mutant in previous studies [30, 31, 34]. In our study, the relative expression of the *Hyp-GALT* genes in the various mutants confirmed the near absence of mutant allele expression in all higher-order mutants (marked with asterisks) along with a concomitant increase or similar amount of expression of the normal alleles (with no mutation). Multiple mutations in the *Hyp-GALT*s generally lead to the upregulation of normal *Hyp-GALT*s for compensation. For example, *GALT8* in *2579* was upregulated up to 2.3-fold in comparison to relative gene expression of 1 in WT.

### *Hyp-GALT* mutants have reduced β-Yariv-precipitable AGPs

To investigate the effect of GALT mutations on glycosylation of AGPs, we performed quantification of β-Gal-Yariv precipitable AGPs. β-Gal-Yariv, a specific binding agent to detect, quantify, and purify AGPs via binding with β-1,3-galactan chains [38]. AGPs were precipitated and quantified from rosette leaf, cauline leaf, stem, roots, siliques, and flowers of *Hyp-GALT* mutant plants.

**Fig. 3:**
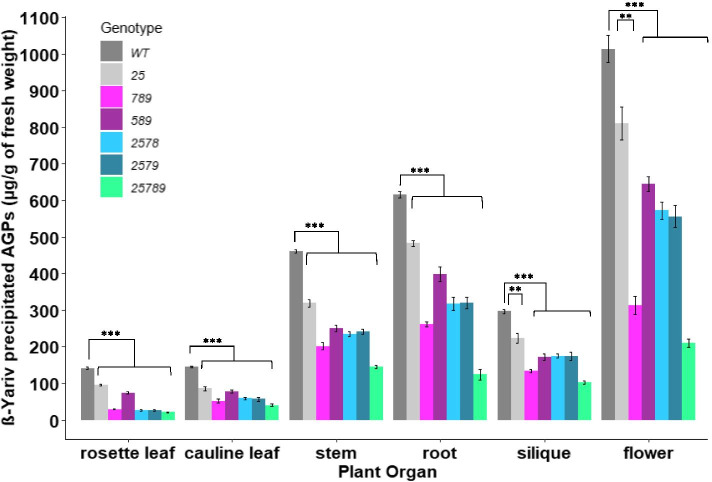
Amount of β-Gal-Yariv precipitated AGPs in WT and higher-order *Hyp*-*GALT* mutants in different organs. All the higher-order mutants showed significant reductions of AGP content in stems, siliques, rosette leaves and cauline leaves. The *25789* quintuple mutants showed the greatest reduction of AGP content in both rosette leaves and stems followed by other double, triple and, quadruple mutants. The asterisks indicate significantly reduced β-Gal-Yariv precipitated AGPs in comparison to the WT controls according to a Student’s t test (**, *P* < 0.01, *** *P* < 0.001; *n* = 4)

Overall, significant reductions in β-Gal-Yariv precipitable AGPs were observed in all organs examined for the mutants (Fig. 3), which is in agreement with the expression patterns of the *Hyp-GALT*s in virtually all plant organs examined. The *25* double mutant produced a 10–42% decrease in precipitable AGP content compared to the WT in various plant organs. In contrast, *789* displayed a much higher reduction in β-Gal-Yariv precipitable AGPs in flowers, roots, and rosette leaves with an average of 69, 59 and 75% reductions respectively compared with WT. The *789* triple mutant also displayed a much greater reduction in AGP content in comparison to the other triple mutant *589*.

The quadruple mutants *2578* and *2579* exhibited nearly similar amounts of reductions of precipitable AGPs such that stem, silique and flowers ranged between 45 and 50%, while cauline leaves and rosette leaves reductions ranged between 60 and 75%. These quadruple mutants *2578* and *2579* did not exhibit AGP reductions as high as in the stems, siliques and flowers of *789*. Introducing mutation of two genes *galt2 galt5* in place of one gene *galt7* in combination with *galt8 galt9* did not produce as much of a reduction in precipitated AGPs as in *789*, indicating that *789* caused a greater effect on AGPs than other GALTs in stem, roots, silique and flower. These results are corroborated by transcript levels depicted by in silico gene expression profiles of *Hyp-GALTs* among different organs where *GALT2* and *GALT5* have lower transcript levels than *GALT7* in carpel and inflorescence (Supplemental Fig. 1). However, the cauline and rosette leaves of *789*, *2578*, *2579* had similar levels of reduction in precipitable AGPs.

Clearly, the *25789* quintuple mutant exhibited the highest reductions in AGP precipitations such that flower, root and rosette leaf exhibited about an 80% decrease whereas other organs (stem, silique and cauline leaf) showed an ~ 70% reduction in precipitable AGPs.

### Monosaccharide composition analysis of the *Hyp-GALT* mutants

**Fig. 4:**
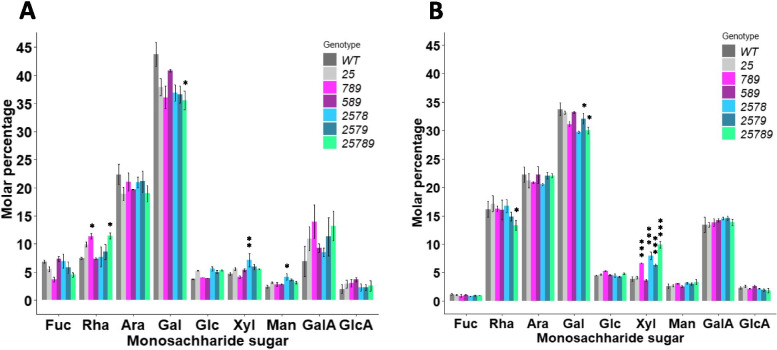
Monosaccharide composition analysis of AGPs isolated from (**A**) silique and (**B**) roots of *Hyp-GALT* mutants. AGPs were extracted and purified from siliques and roots of *Hyp-GALT* mutants and WT plants (*n* = 3). An analysis of variance (ANOVA) on these mutants yielded significant variation among genotypes. A post hoc Tukey test was applied to see which groups were significantly different from WT (Col-0). *P < .05, **P < 0.005, ***P < 0.001

To investigate the effect of introducing *Hyp-GALT* mutations on AGP sugar compositions, β-Gal-Yariv purified AGPs were extracted from silique and root tissues of *Hyp-GALT* mutants and were subjected to monosaccharide composition analysis (Fig. 4). The data showed that AGPs from all organs were mainly composed of Gal and Ara residues in approximately 1.5–2:1 molar ratio. AGPs obtained from the roots and siliques generally demonstrated a decrease in Gal content in the *Hyp-GALT* mutants compared to the WT.

The *25789* mutant siliques displayed a maximum reduction in Gal content of 12.5% in siliques (Fig. 4A) and 18.6% in roots (Fig. 4B). For silique tissues, *25789* was followed by less severe Gal content reductions in the three lesser mutants (*789, 2578* and *2579*); while *589* and *25* showed Gal content similar to WT. In root tissues, *25789* was followed by smaller reductions in Gal content in four mutants (*25*, *789, 2578* and *2579*); while *589* showed Gal content similar to WT. Unlike roots, the Rha in siliques also decreased significantly in higher-order mutants specifically in *25789*. A concomitant increase in the percentages of other sugars like Xyl and/or Man were observed in root and silique with subtle variations, which is likely a result of expressing the data as molar percentages.

**Fig. 5:**
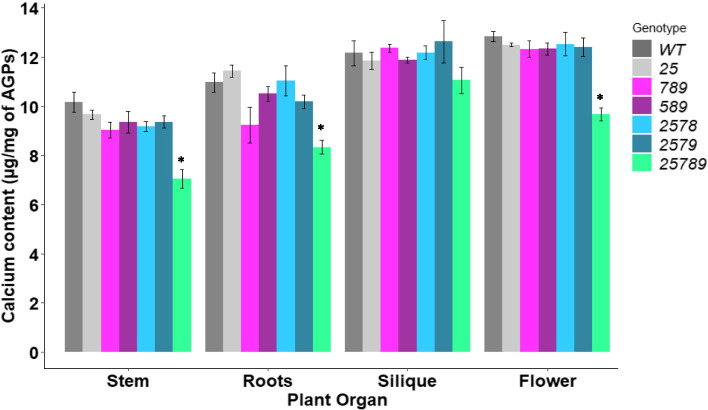
Calcium binding ability of AGPs of *Hyp-GALT* mutant stem, roots, siliques, and flowers. AGPs were extracted and purified from stem, flower, silique and roots of *Hyp-GALT* mutants and WT plants (*n* = 3). Student’s t-test was employed to calculate significant differences from respective WT samples of each organ. (*P < 0.05, **P < 0.01, ***P < 0.001)

Interestingly, the calcium content bound to extracted AGPs displayed a significant reduction (12–31%) in the silique, flower, stem and root tissues of the quintuple mutant *25789* only but not in any other *Hyp-GALT* mutant compared to the WT (Fig. 5).

### *Hyp-GALT* mutants exhibit germination defects

**Fig. 6:**
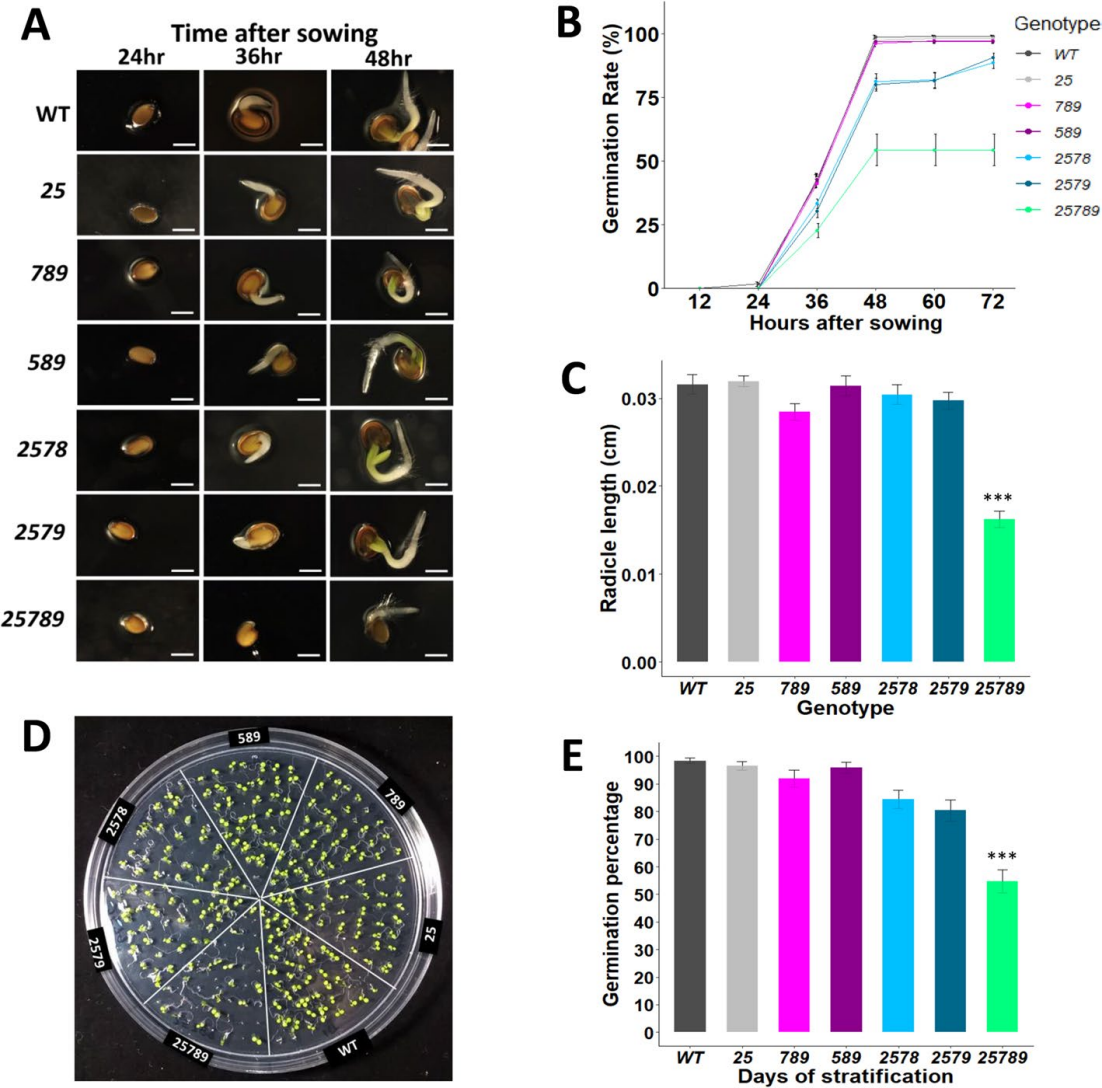
Germination rates and percentages of *Hyp-GALT* higher-order mutants. **A** Representative images for the germination rate as indicated by the emergence of the radicle from the seeds of the WT and higher-order *Hyp-GALT* mutants on ½ MS media. In comparison to WT seeds, the mutants displayed delayed germination. Seeds of all the genotypes were sterilized, plated on ½ MS media, and then stratified for 3 d. Images were taken at 12, 24, 36, 48, 60 and 72 h after sowing. Scale bar = 0.5 mm. **B** Germination rates were measured in three independent experiments (*n* = 96). Radicle length was measured by Motic Image version 3.2 at 48 h and quantified in (**C**). For radicle length, 35 germinated seeds from each genotype were measured with three biological experimental replicates. An analysis of variance (ANOVA) on these mutants yielded significant variation in radicle lengths. A post hoc Tukey test was applied to see which groups were significantly different from WT (Col-0). *P < .05, **P < 0.005. **D** Germination percentages of the higher-order *Hyp-GALT* mutants. Photos of the seedlings of *Hyp-GALT* mutants and WT (WT) at 4 DAG were taken, and germination percentages were determined and compared to WT (control) plants under normal conditions (*n* = 144) in four independent experiments (**E**). An analysis of variance (ANOVA) on these mutants yielded significant variation among genotypes. A post hoc Tukey test was applied to see which groups were significantly different from WT (Col-0). *P < .05, **P < 0.005, ***P < 0.001. DAG, days after germination

A significant delay in germination of the quintuple *25789* was observed under normal conditions in post-harvested (> 6–8 months) seeds. At 36 h, only 22% germination was seen in the quintuple *25789* mutant, followed by quadruples, *2578* and *2579* demonstrating 30–33% germination rates in comparison to WT, while *25, 589* and *789* exhibited maximum germination rates (40–43%) similar to WT. By 48 h, all genotypes germinated to their maximum germination percentages. At 48 h, the quintuple *25789* mutant showed a maximum germination rate of 54% whereas quadruples *2578* and *2579* displayed a maximum germination rate of 80–83% in comparison to WT, *25, 589* and *789*, which attained 95–98% germination (Fig. 6A and B).

Furthermore, the radicle length was measured at 48 h after sowing. The *789* mutant showed a slightly negative effect on radicle length growth, though statistically non-significant. Interestingly, the quintuple mutant *25789* showed a significantly smaller radicle length (41% smaller radicle length) compared to the WT and all other *Hyp-GALT* mutants (Fig. 6C). In addition, for the post-harvested (> 6-8 months) seeds, germination percentages reduced in quadruple (*2578, 2579*) by 12 and 15% which were not statistically significant though. The quintuple (*25789*) mutants showed significant decrease of 45% in germination percentage (Fig. 6D and E) in comparison to WT. For the WT, *25, 789,* and *589,* the germination percentage ranged between 94 and 99%.

### *Hyp-GALT* mutants exhibit stunted plant growth when grown on soil and plates

**Fig. 7:**
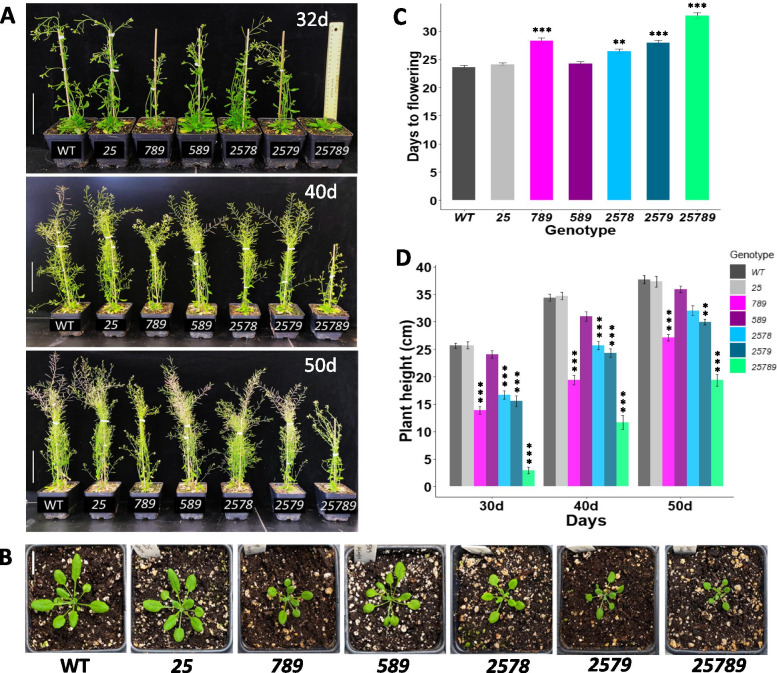
Aerial growth phenotypes of the *Hyp-GALT* mutants under normal conditions. **A** Growth phenotypes of the *Hyp-GALT* mutants and WT grown in soil at 32, 40 and 50 DAG and compared to WT plants. Scale bar = 10.0 cm. **B** *Hyp-GALT* mutants along with WT growing on soil at 21 DAG. Scale bar = 1.0 cm. **C.** Quantification of days to flowering in the *Hyp-GALT* mutants and WT grown in soil. **D** Plant height quantified at 32, 40, and 50 DAG and compared to WT (control) plants under normal conditions (*n* = 15) in three independent experiments. Analysis of variance (ANOVA) on these mutants yielded significant variation among conditions. A post hoc Tukey test was applied to determine which groups were significantly different from WT. *P < .05, **P < 0.005, ***P < 0.001. DAG, days after germination

**Fig. 8:**
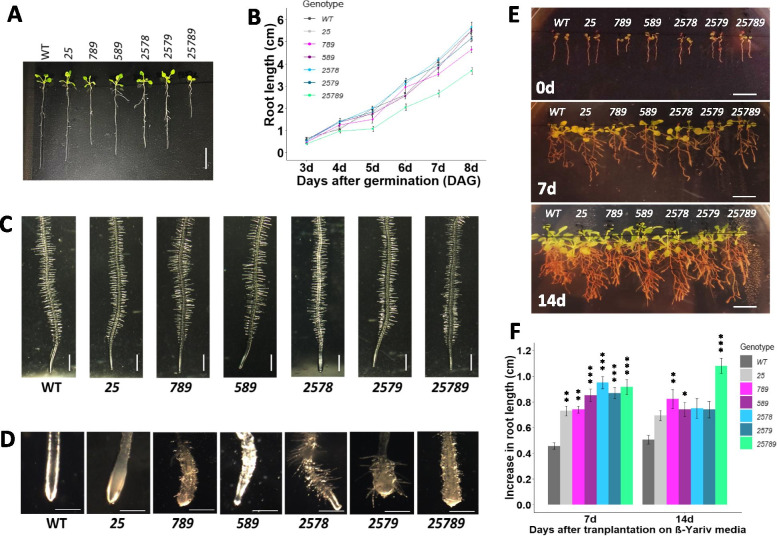
Root growth phenotypes of the *Hyp-GALT* mutants under normal and stress conditions. **A** Seedlings of the *Hyp-GALT* mutants and WT grown vertically on ½ MS media at 8 DAG. Scale bar = 1.0 cm. **B** Root growth curves of the *Hyp-GALT* mutants grown on ½ MS media were measured from 3 to 8 DAG and compared to WT under normal conditions (*n* = 15) in three independent experiments. **C** Root-hair length and root-hair density of seedlings of the *Hyp-GALT* mutants and WT grown on ½ MS media at 10 DAG. Scale bar = 1.0 mm. **D** Salt-induced root anisotropic growth defects in the *Hyp-GALT* mutants. Light microscopic images of root tips of plant seedlings of indicated genotypes grown in ½ MS supplemented with 100 mM NaCl at 10 DAG. Seeds were germinated in ½ MS plates and grown for 3 d before transferring to MS plates with 100 mM NaCl. Scale bar = 1.0 mm. **E** Reduced inhibition of primary root growth of the *Hyp-GALT* mutants in the presence of β-Gal Yariv reagent**.** Root lengths were measured at 7 d and 14 d after transfer of seedlings from ½ MS plates at 4 DAG, onto ½ MS plates supplemented with 50 μM β-Gal Yariv reagent and quantified in (**F**). Statistical differences were determined by one way ANOVA, followed by the Tukey’s honestly significant difference test. Asterisks represent the statistical significance between genotypes (*, *P* < 0.05; **, *P* < 0.01; ***, *P* < 0.001) within a treatment group in comparison to WT. Vertical bars represent mean ± SE of the experimental means from at least three independent experiments (*n* = 15 seedlings). DAG, days after germination

To investigate the effect of *Hyp-GALT* mutations on plant vegetative growth, mutants and WT were grown on ½ MS media. The higher-order *Hyp-GALT* mutants exhibited pronounced pleiotropic morphological alterations in vegetative growth and bolting (Figs. 7 and 8) whereas single *Hyp-GALT* mutants showed no obvious phenotypes in previous studies [31, 32, 34]. The *789* triple mutant, as well as the *2578* and *2579* quadruple mutants showed significant reductions in plant height by 14–25%, while the quintuple mutant *25789* demonstrated 40% reduction in plant height compared to WT at 50 DAG **(**Fig. 7A and D**)**. The *589* triple and *25* double mutants, however, did not show any significant effect with respect to this growth phenotype.

The days to flowering was also delayed significantly in the *789*, *2578* and *2579* mutants by 3–5 d and in the quintuple mutant *25789* by 10–11 d (Fig. 7A and C). The higher-order *Hyp-GALT* mutants also demonstrated retarded growth of rosette leaves as seen in Fig. 7B; this is evident in *789*, *2578*, *2579*, and is the most severe in *25789* (Supplemental Fig. 3).

The *2578* and *2579* quadruple mutants showed no significant primary root growth. The *789* mutants showed significant reduction (21%) in primary root growth while the *25789* quintuple mutants displayed the most retarded primary root growth (38%) **(**Fig. 8A and B**)**. In contrast, the root hair density and root-hair length were not affected much **(**Fig. 8C and Supplemental Fig. 4) in these mutants with the exception of the *25789* mutant which showed a small increase in root hair density and a small decrease in root hair length.

The higher-order *Hyp-GALT* mutants showed much more pronounced salt hypersensitivity response in form of root tip swelling compared to previously reported in single and double *Hyp-GALT* mutants [34]. All of the higher-order *Hyp-GALT* mutants displayed root tip swelling and decreased root elongation compared to WT plants; however, *589, 2578, 2579* showed the less severe root growth defects in comparison to *789* and *25789* (Fig. 8D and Supplemental Fig. 5). β-Yariv reagent is known to inhibit root growth by binding to AGPs [8, 35, 39]. Seedlings of all genotypes were grown in the presence of β-Gal-Yariv reagent such that WT seedlings displayed reduced root growth as expected (Fig. 8E). In contrast, all *Hyp-GALT* mutants showed a β-Yariv insensitive root growth phenotype; *789* and *25789* displayed the highest β-Yariv insensitivity with respect to root growth (Fig. 8F) as quantified 7d and 14d after transfer to β-Yariv.

### Altered cell wall structure in *25789* quintuple mutant stems

**Fig. 9:**
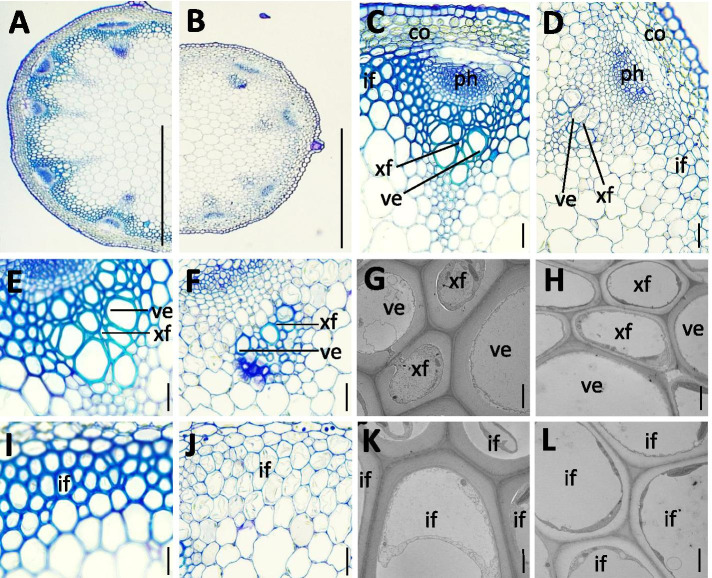
Altered stem cell wall architecture in *25789* mutant. **A** and **B** Cross section of the WT (**A**) and *25789* mutant stem (**B**). **C.** and **D.** Cross section of the vascular bundle region of WT (**C**) and *25789* mutant (**D**). **E.** and **F.** Cross sections of vascular bundles of WT (**E**) and *25789* mutant (**F**). **I.** and **J.** Cross section of interfascicular region of WT (**I**) and *25789* mutant (**J**). **G** and **H** Transmission electron micrographs of xylem cells of WT (**G**) and *25789* mutant stem (**H**). **K** and **L.** Transmission electron micrographs of interfascicular fiber cells of WT (**K**) and *25789* mutant stem. co: cortex, if: interfascicular fiber, ph: phloem, ve: vessel, xf: xylary fiber. Scale bars = 500 μm in (**A**, **B**), 20 μm in (**C**, **D**, **E**, **F**, **I** and **J**), 2 μm in (**G**, **H**, **K**, **L**)

Since the *25789* mutants exhibited stunted seedling growth and shortened inflorescence stems **(**Fig. 7**)**, transverse sections of fresh tissue from the base of the 8-week-old inflorescence stem were stained with toluidine blue for cell wall polysaccharides, to visualize differences in primary and/or secondary cell wall morphology (Fig. 9). The results revealed that *25789* mutant stems **(**Fig. 9A, B**)** have smaller vascular bundles **(**Fig. 9C, D**)** with reduced thickness of fiber cells, xylem fibers, vessels and interfascicular fibers stems **(**Fig. 9F, H**)** compared to the WT **(**Fig. 9E, G**)**. Moreover, transmission electron microscope (TEM) analysis of cross sections confirmed thinner secondary cell walls in the vessels, vascular fibers and interfascicular fibers of the *25789* mutants **(**Fig. 9H, L**)** relative to WT **(**Fig. 9G, K**)**.

### *Hyp-GALT* mutants display reduced seed set and abnormal seed morphology

**Fig. 10:**
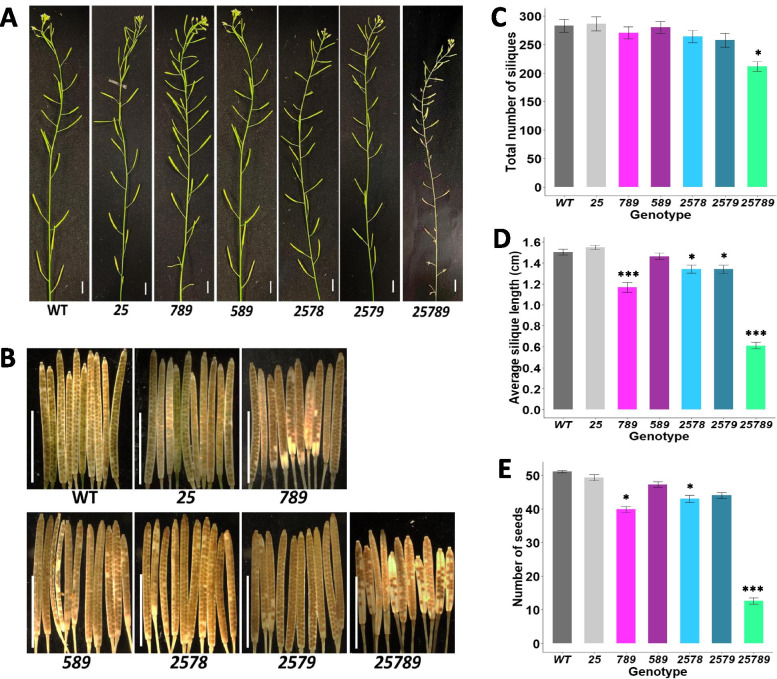
Seed set for the *Hyp-GALT* mutants. **A** Representative inflorescences of the higher-order *Hyp-GALT* mutants and WT at 42 DAG. Scale bar = 1.0 cm. **B** Representative tenth siliques from the base of each higher-order *Hyp-GALT* mutant and WT grown on soil. Scale bar = 1.0 cm. **C** Total number of siliques in each genotype (*n* = 15) at 60 DAG. Data shown represent the mean values of three biological replicates ±SE. **D** and **E** Quantification of the silique lengths (**D**) and seed fill (**E**) in WT and *Hyp-GALT* mutants. The fifteen siliques (starting from the base) from of all higher-order mutants used were quantified with respect to silique length and seed set in Supplemental Figs. S7 and S8. Data presented here are means ± standard error (SE) (*n =* 150 for silique length and *n* = 300 for seed-set). Statistical differences were determined by one way ANOVA, followed by the Tukey’s honestly significant difference test. Asterisks represent the statistical significance between genotypes (*, *P* < 0.05; **, *P* < 0.01; ***, *P* < 0.001). DAG, days after germination

The higher-order *Hyp-GALT* mutants displayed a significant reduction in total siliques per plant in the *25789* mutants (25%) in comparison to WT unlike the other mutants **(**Fig. 10C**).** The *25789* mutant also demonstrated most dramatic reduction in seed set (~ 70%) compared to 14–15% reductions in the average seed set for the *789, 2578, 2579* mutants*;* **(**Fig. 10A, B and E**)**. The analysis of basal (oldest) fifteen siliques on the main inflorescence stem also showed the reduction in seed set of *25789* mutant compared to the WT **(**Fig. 10A and S6). The average silique length was also reduced in the higher-order *Hyp-GALT* mutants **(**Fig. 10A, B and D**)**. The average silique length was affected more for *789* (22% reduction) than both *2578* and *2579,* which showed 11% reductions; however, *25789* exhibited a 60% reduction (Fig. 10E and Supplemental Fig. 7).

**Fig. 11:**
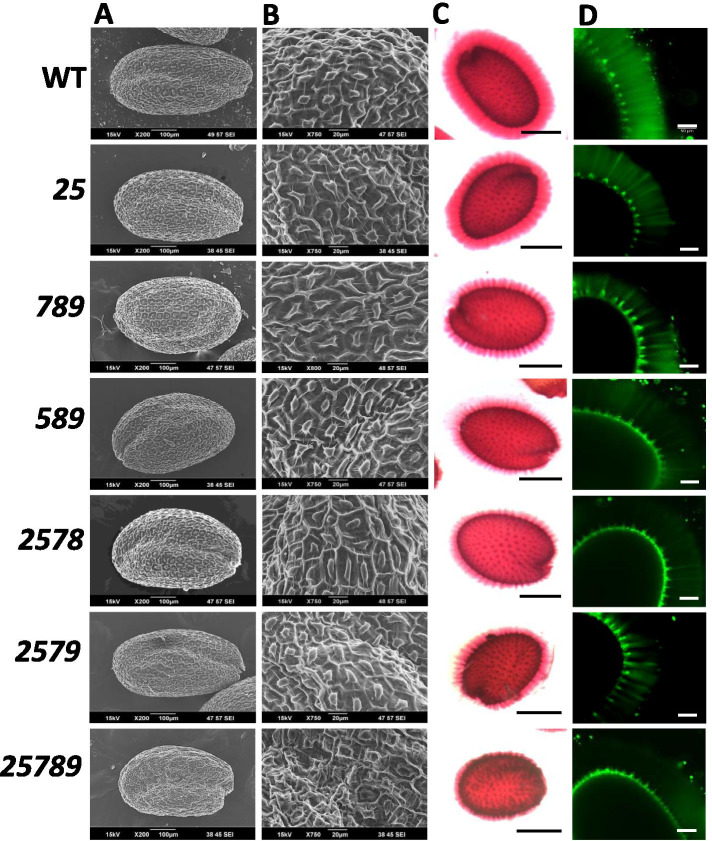
Seed morphology and seed mucilage phenotypes of the higher-order *Hyp-GALT* mutants. **A** and **B** Scanning electron micrographs showing whole seed and seed coat details of higher-order *Hyp-GALT* mutant and WT seeds. Note the hexagonal epidermal cells with thickened radial cell walls and volcano-shaped columellae in the center of each cell. Scale bar = 100 μm (**A**) and 20 μm (**B**). **C** Staining of seed coat mucilage for pectin with ruthenium red following the removal of the outer mucilage in three independent experiments (*n* = 30). Scale = 0.2 mm. **D** Staining of seed coat mucilage for cellulose and other β-glucans with Calcofluor white stain. The stain labels the columella, tangential cell wall remnants and rays deposited across the inner layer of mucilage of the hydrated seeds. Scale bars = 50 μm

SEM seed morphology analysis revealed that the quintuple mutant exhibited altered seed shape and disfigured remnants of columellae. No such discernable differences were found in other mutant seeds **(**Fig. 11A and B). To examine the involvement of AGPs and the *Hyp-GALTs* in modifying seed coat mucilage, ruthenium red staining which stains acidic biopolymers such as pectin, and calcofluor staining, which stains cellulose as well as β-glucans, were done with the *Hyp-GALT* mutants **(**Fig. 11C and D). All *Hyp-GALT* mutants displayed reduced cellulose ray staining and reduced pectin staining in the mucilage adhering to the seeds compared to the WT with *25789* displaying the strongest reduction in seed mucilage pectin and cellulose staining. To examine and quantify the alterations in the outer, non-adherent mucilage versus the adherent mucilage, sequential extraction of seeds with ammonium oxalate and 0.2 N NaOH (for extraction of soluble and weakly attached pectins) followed by 2 N NaOH (for extraction of strongly linked pectins and cross-linking glycans/hemicelluloses) was performed [40] (Supplemental Table 4). The higher-order *Hyp-GALT* mutant seeds had a significant increase in the total sugar present in the ammonium oxalate and 0.2 N NaOH extracts with a concomitant decrease in the adherent mucilage compared to WT seeds. These observations corroborate the results of ruthenium red staining which also suggested a decrease in adherent mucilage of the higher-order *Hyp-GALT* mutants.

### *Hyp-GALT* mutants display anther and pollen defects

*Hyp-GALT* genes are highly expressed in the inflorescence and in the pollen (Supplemental Fig. 1). Moreover, as previous studies on single *Hyp-GALT* mutants also demonstrated pollen tube growth defects [31], we microscopically examined our higher order *Hyp-GALT* mutants for phenotypic differences in male reproductive tissues. As shown in the Supplemental Fig. 8, the arrangement of floral reproductive organs appeared indistinguishable from the WT plants, although the overall flower size was smaller in the *25789* mutant.

**Fig. 12:**
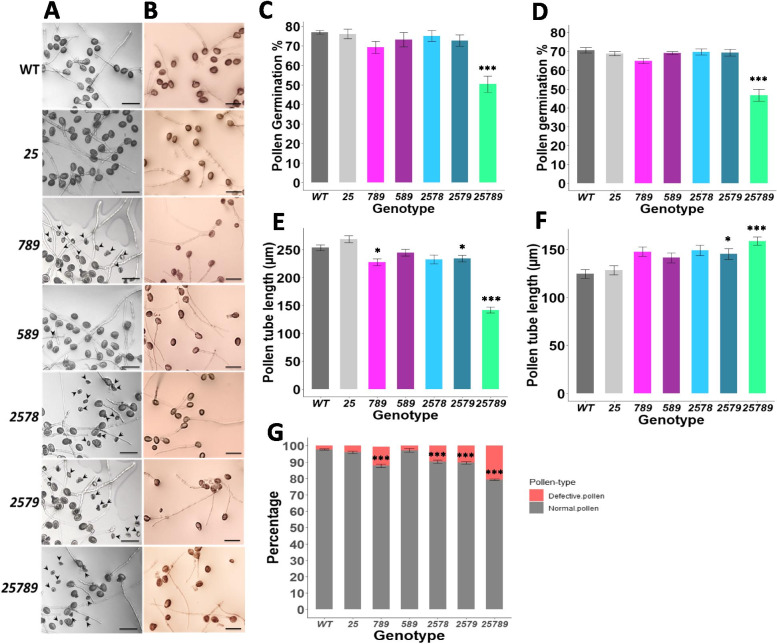
Pollen phenotypes of the higher-order *Hyp-GALT* mutants. **A** and **B** Representative images of in vitro pollen assay showing pollen tubes from *Hyp-GALT* mutants in pollen germination medium after 3 h (column A) and pollen germination medium supplemented with 30 μM β-Gal Yariv after 1.5 h (column B). Scale bar = 100 μm. **C** and **E** represent the pollen germination percentages and pollen tube lengths of *Hyp-GALT* mutants in pollen germination medium. **D** and **F** represent the pollen germination percentages and pollen tube lengths of *Hyp-GALT* mutants in pollen germination medium supplemented with 30 μM β-Gal Yariv. For each measurement made in **B**, **C**, **D**, and **E**, twenty flowers of each genotype and 5 pollen tubes for each flower were measured using Image J. The experiment was repeated five times (total *n* = 500), and the values were subjected to statistical analysis by ANOVA, followed by the Tukey’s honestly significant difference test. (*, *P* < 0.05; **, *P* < 0.01; ***, *P* < 0.001). **G** The in vitro pollen germination assay showed defective pollen (indicated by arrows) in higher-order *Hyp-GALT* mutants compared to WT (**A**) and percent defective pollen in each genotype was calculated and quantified in (**G**)

**Fig. 13:**
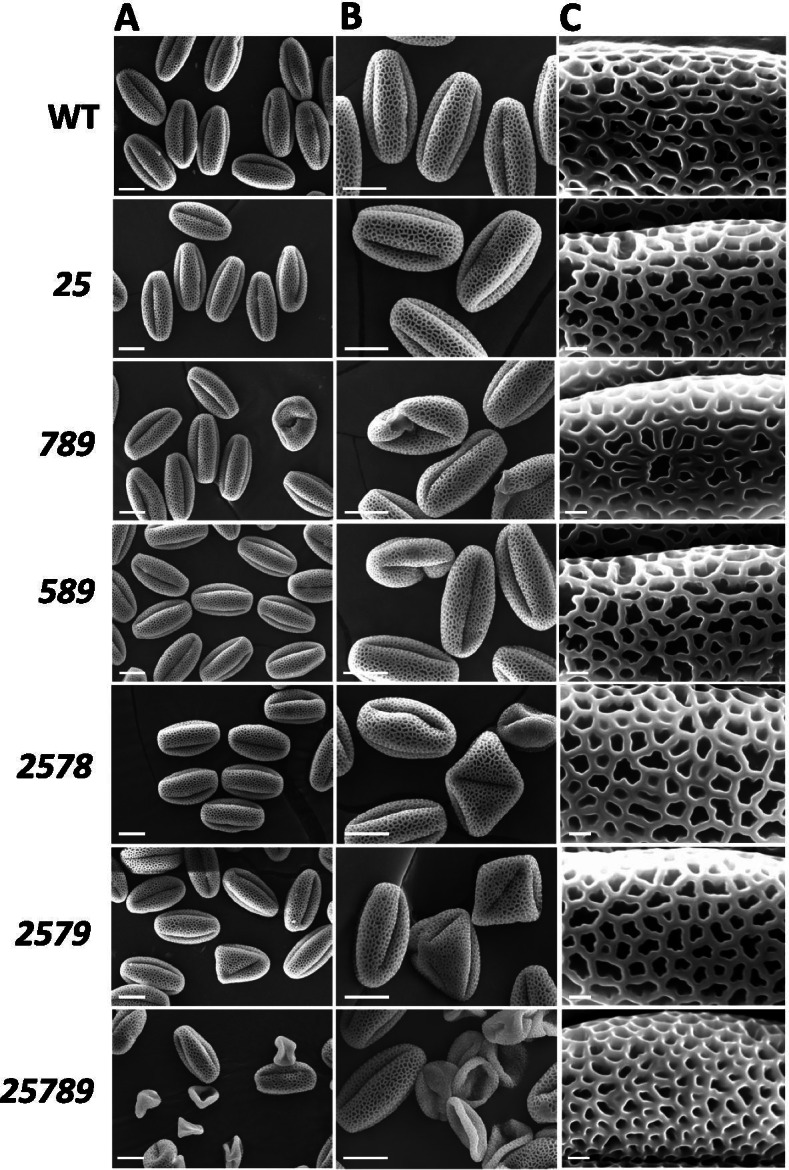
Scanning electron micrographs of pollen grains of WT and the higher-order *Hyp-GALT* mutants. **A**, **B**, and **C** columns represent the pollen grains of WT, *25*, *789*, *589*, *2578*, *2579*, and *25789* at different magnifications. Genotypes WT, 25, and 589 genotypes show the regular reticulate exine structure details of exine whereas *789*, *2578*, and *2579* show some misshaped and abnormal pollen. *25789* pollen grains demonstrated a higher percentage of collapsed and defective pollen with abnormal exine structure with smaller lacunae. Scale bars = 10 μm in column **A** and **B**, 1 μm in **C**

In vitro pollen germination was affected only for *25789*, which showed 50% germination in comparison to 77% germination in the WT (Fig. 12A and C). For pollen tube lengths, an 8–10% reduction was observed for mutants, *789, 2578,* and *2579,* whereas *25789* exhibited a 44% reduction compared to WT (Fig. 12DD). A defective pollen phenotype was also observed in the *Hyp-GALT* mutants (Figs. 12G and 13)*.* Around 10–12% of defective pollen was observed in the *789, 2578,* and *2579* mutants*,* whereas the *25789* mutant exhibited 20% defective pollen. Further, SEM analysis revealed defects in *25789* with abnormal exine structure with smaller lacunae and abnormal reticulate structure (Fig. 13).

The *789, 589, 2578, 2579,* and *25789* mutants also displayed reduced inhibition of pollen tube growth in response to β-Gal-Yariv reagent compared to WT (Fig. 12B, E and F); maximum pollen tube elongation was observed in *25789* which was 44% longer than WT.

## Discussion

### Gene structure, expression and functions of two small *Hyp-GALT* multigene families

Eight *Hyp-GALT* genes belonging to two small multigene families (clades) in the CAZy GT31 family are known to add galactose onto the peptidyl Hyp residues in AGP core proteins This addition represents the first committed step in AG polysaccharide addition and an ideal control point to study the importance and contributions of Hyp-linked type-II AG chains to the biological functions of AGPs. In addition to the conserved GALT domain in all Hyp-GALTs, the GALT2–6 family members in clade B (subclades V and VI) contain a GALECTIN domain, which is absent in the GALT7–9 family members in clade A (subclade III) of the GT31 phylogenetic tree for Arabidopsis [19]. These structural domain differences attributed to the GALECTIN domain (Fig. 1A) might indicate that the two subclades of β-(1,3)-GALTs may have multiple and distinct functions in *O-*glycan biosynthesis compared to the other subclades which lack this domain. Until now, the function of galectin domain in these subclades is not known but all the members of these two subclades are known to add first galactose to Hyp residue of AGP protein backbones. Indeed, one member of this galectin-containing subclade V, *GALT1* (*At1g26810*) was shown to encode a (1,3)-β-D-GalT involved in the biosynthesis of the Lewis a epitope on N-glycans [19, 41]. Arguably, galectin domain might help in holding another galactose after the first one for β-(1,3)-GalT linkage, but further studies are required to reveal their exact mechanism of action. Thus, subgroup of galectin containing β-(1,3)-GalTs may have multiple and distinct functions in both N-glycan and *O*-glycan biosynthesis.

Functional redundancy of both *Hyp-GALT* families is consistent with their substantial and widespread expression in almost all organs and growth stages, indicating the importance of these genes for variety of physiological functions in both vegetative and reproductive growth and development. Moreover, in previous studies prominent phenotypes were not reported for the single or double mutants [30, 31, 34], while the *789* triple knockout mutant exhibited various morphological alterations both in roots and aerial tissues [32]. Consequently, this study produced a set of higher-order knock-out mutants for five of the *Hyp-GALT* genes, including a quintuple mutant, followed by molecular, biochemical, and physiological characterization of these mutants to dissect the functional roles of each of these GALTs with respect to AGP biosynthesis and function in various plant organs. Upregulation of the rest of *Hyp-GALT* WT alleles in these multiple gene knock-outs is likely the direct consequence of gene compensation to mitigate the loss of Hyp-GALT protein functions (Fig. 2).

### Higher-order *Hyp-GALT* mutants have less glycosylated AGPs with reductions in gal and calcium content in various organs

Our results showed that higher-order *Hyp-GALT* mutants exhibited significant decreases in AGP content of flower, stem, root, silique, rosette leaf and cauline leaf in comparison to WT (Fig. 3). The greatest decrease in AGPs was seen in the *25789* mutant (70–80%) and included all organs which were examined, indicating that these *Hyp-GALT* genes function redundantly in AGP glycosylation in these organs. These findings are consistent with previous results that showed the *25* double mutants displayed a 43% reduction in β-Gal-Yariv precipitable AGPs compared to WT [33]. However, we only observed a 70% decrease in precipitable AGPs int the *789* triple mutant, whereas a 90% decrease was reported previously [32]. Furthermore, monosaccharide composition analysis supported and extended the above results by showing a reduction in Gal content in silique and roots in almost all higher-order *Hyp-GALT* mutants **(**Fig. 4**)**.

A significant reduction in calcium content for the *25789* mutant only was observed (Fig. 5**)**. AGPs, through their terminal GlcA residues, can interact with other cell wall components and/or with calcium to play roles in wall crosslinking and cellular signaling [42, 43]. Indeed, there are number of studies that provide evidence for AGP-AGP crosslinking as well as AGP-pectin and AGP-pectin-arabinoxylan crosslinking [44–46]. Most notably, Arabinoxylan Pectin Arabinogalactan Protein 1 (APAP1) is one such complex in which AGP57C is chemically cross-linked to pectin and hemicellulose (i.e., arabinoxylan). Moreover, the GlcA residues on this AGP are known to be involved in the chemical crosslink to pectin [45]. A recent study by Lopez-Hernandez et al. [47] demonstrated that AGPs act as a calcium capacitor to bind and release cell-surface apoplastic calcium in a pH-dependent manner. Here, the *Hyp-GALT* mutants, particularly the *25789* mutant, exhibited reduced/aberrant AG-polysaccharides which might have a reduced GlcA linking to calcium. Such biochemical alterations are clearly correlated and may be responsible for the impaired physiological phenotypes/functions observed here related to plant growth, development and reproduction.

### AGP glycosylation is critical for normal growth and development

Higher-order *Hyp-GALT* mutants exhibited pleiotropic effects on vegetative growth under normal growth conditions, such that plant height, rosette leaf size, primary root growth and root hair length all decreased, while days to flowering and root hair density increased. Such growth defects were not observed for single or double mutants but were present in *789* triple mutants to some extent, indicating partial gene redundancy between the different *GALTs*. Taken together with our biochemical data, it is reasonable to conclude that critical threshold levels of glycosylated AGPs are required for normal growth and development.

AGPs have been implicated in various aspects of plant growth and development including cell proliferation and elongation, pattern formation, programmed cell death, cell-cell communication and hormone signaling [6, 11, 48, 49]. Several AGP and AGP-related GT mutants are known to display such pleiotropic growth defects. For example, an *agp19* mutant demonstrated smaller rosette leaves, delayed growth, shorter hypocotyls and inflorescence stems, and fewer siliques with less seeds [50]. Root hair sensitivity was previously observed in mutants for prolyl-4-hydroxylase genes (P4H2, P4H5, P4H13), which displayed impaired root hair growth [51, 52]. Additionally, AGP30, a non-classical histidine-rich AGP is strongly expressed in roots and *agp30* mutant revealed its function in in vitro root regeneration and in planta timing of seed germination [53] while AGP31 is strongly expressed in vascular tissues including the phloem and primary xylem and is suggested to play a role in root development [54]. Hence, knocking out multiple *GALT* genes likely affects such AGPs resulting in under-glycosylated AGPs, which in turn can prevent these AGPs from properly interacting with other cell wall or plasma membrane components in a structural or signaling capacity. In other words, our work is beginning to elucidate the critical roles that the carbohydrate moieties decorating AGPs play in normal growth and development.

### AGP glycosylation is important for root cell expansion and growth under stress conditions

All *Hyp-GALT* mutants display root-tip swelling in response to salt stress. These findings are supported by the higher levels of expression for *GALT7, GALT8* and *GALT9* compared to *GALT2* and *GALT5* in the roots (Supplemental Fig. 1). However, more pronounced root tip swelling was found in *789* and *25789* in comparison to *25, 2578,* and *2579*, and which could be recovered in *25, 2578,* and *2579* unlike *789* and *25789* (Supplemental Fig. 5) This argues for the stronger involvement of *GALT7, GALT8* and *GALT9* in root growth. Previously, similar reduced root growth in the presence of elevated NaCl was also observed in the *FUT4* and *FUT6* mutants, which are defective in AGP fucosylation [26, 27]. Our root swelling observations with the *Hyp-GALT* mutants coincide with and extend previous observations by [34]. We now believe that the GALT2/GALT5-dependent glycosylation of an SOS5 AGP which might act as cell wall integrity sensor for perceiving and signaling differences in turgor pressure when subjected to elevated salt, can be extended to include other *GALT*s, namely *GALT7, GALT8* and *GALT9*.

All the higher order *Hyp-GALT* mutants displayed stronger insensitivity of the roots to β-Gal-Yariv growth inhibition. These results are in agreement with previous findings that single *galt 2–6* mutants, *galt2 galt5* double mutant, *galt7 galt8 galt9*, *galt3 galt4 galt6* triple mutants and *galt2 galt3 galt4 galt5 galt6* quintuple mutants were shown to be less sensitive to β-Yariv inhibition of root growth [31, 35]. In our study here, *25789, 2579, 2578* higher order mutants exhibited the greatest root elongation in comparison to the double and triple mutants, indicating the partial redundancy of *Hyp-GALTs*. The fact that β-glycosyl-Yariv reagent inhibits cell proliferation by binding to AGPs and forming insoluble complexes or can even lead to cell death explains the lesser growth inhibitory effects experienced in mutants due to reduced or under glycosylated AGPs.

### Altered AGP glycosylation affects cell wall structure

Plant mechanical strength primarily depends on cell wall composition. Although AGPs represent < 10% of the complex network of cell wall polysaccharides, multiple studies have reported their effect on mechanical strength by modifying and customizing the synthesis and architecture of other major components and hence, maintaining the integrity of the cell wall. The *25789* quintuple mutants exhibited thinner stem xylem fibers, vessel and interfascicular fiber cell walls compared to WT. These cell wall defects are similar to what has previously been observed in group A fasciclin-like AGPs of Arabidopsis, *fla11fla12* double mutants that displayed reduced tensile strength and stiffness, an increase in cellulose microfibril angle, reduction in cellulose, galactose and arabinose content and an effect on stem biomechanics [55]. Another mutant belonging to group B fasciclin-like AGPs, *fla16* exhibited moderate glycosylation and reduced stem length, also demonstrated its role in plant secondary cell wall synthesis and function [56]. Moreover, a brittle stem and zebra leaf mutant *bz1* of rice which encodes a functional UDP-galactose/glc epimerase exhibited a significantly decreased AGP levels, and also displayed reduced mechanical strength along with altered cell wall structure and composition [57]. This supports the idea that AGPs somehow interact with the scaffold proteins that align microtubules and cellulose microfibrils, eventually disorganizing their orientation [58–60]. Although the details about how AGPs structurally affect other cell wall components remain unclear, the monosaccharide composition analysis along with the changes in cell wall structure indicates that under-glycosylated AGPs in the *Hyp-GALT* mutants alter the cell wall architecture.

### AGP glycosylation affects seed germination, morphology and mucilage accumulation

Germination rates of seeds were reduced for the *2578* and *2579* mutants, but the germination percentage was reduced dramatically only for *25789* (∼55–60% germination) (Fig. 6). The germination defects are consistent with the higher gene expression of these five *GALT* genes in seed coat differentiation during early embryogenesis (Supplemental Fig. 2). SEM images of dry, WT seeds display an epidermal layer consisting of hexagonal cells, thickened radial cell walls and a raised structure known as the columella. In the mutants, the columellae of the seed epidermal cells are irregular in shape and reduced in size. Seed development has two major phases, embryo development and seed maturation [61]. Thus, *25789*’s failure to achieve complete germination may be due to irreparable damage caused to the embryo during embryogenesis or to structural alterations in the *25789* seed coats during the dry storage of seeds (Fig. 11A).

Unlike WT seeds, the seeds of higher-order *Hyp-GALT* mutants extrude a reduced amount of adherent mucilage upon hydration, which was more pronounced than observed in *25* and *789* (Fig. 11). In addition**,** all the mutants show a substantial reduction of cellulosic rays in the seed coat mucilage layer. Although AGPs represent a small portion of the seed coat mucilage, they play important roles [62]. Harpaz-Saad et al., (2011) [40] reported that synthesis and deposition of cellulosic rays in seed coat mucilage were affected by both FEI2, a leucine-rich receptor-like kinase, and SOS5, a fasciclin-like AGP, with a concomitant increase in solubility of the pectin. Thus, our results indicate that glycosylated AGPs are involved in maintaining the adherent mucilage layer in seeds, most likely through interactions with other mucilage polysaccharides like pectin.

### AGP glycosylation reduces seed yield and affects male gametophyte

The seed set, silique length and total number of siliques were reduced in various higher-order *Hyp-GALT* mutants accompanied by the defective pollen, reduced anther size, and pollen tube lengths **(**Figs. 10 and 13**)**. Also, β-Yariv induced growth inhibition for pollen tubes in *Hyp-GALT* mutants similar to that observed in the roots. The *Hyp-GALT* mutants phenocopied *kns4* mutants*,* which are defective in the *KNS4* gene encoding a type II arabinogalactan β-(1,3)-galactosyl-transferase in the GT31 family and showed reduced fertility attributed to aborted pollen having an abnormal pollen exine structure [20, 63]. Indeed, several AGP genes show high expression in hydrated or mature pollen and pollen tubes in Arabidopsis, including AGP6, AGP11, AGP15, AGP21, AGP22, AGP23, AGP24 and AGP40 [12]. Heterozygous *agp6 agp11* knockouts of two pollen-specific functionally redundant genes, *AGP6* and *AGP11,* had fewer seeds per silique, which was attributed to a reduction in pollen germination as well as pollen tube length [9]. It is proposed that AGPs in the cell wall of pollen tubes can be recycled to maintain their concentration levels at the pollen tube apex, as this may be necessary for them to perform a signaling role for pollen tube guidance [11, 42, 64]. Also, AGPs affect the structure of the cell wall by changing the localization of other cell wall components, suggesting their critical role in the mechanical properties of the pollen cell wall [65]. All *GALTs* examined here, namely *GALT2, GALT5, GALT7, GALT8* and *GALT9*, are likely involved in the glycosylation and hence the function, of pollen specific AGPs.

Clearly, with respect to the mechanism of action of AGPs in reproduction, the interactive sugar surfaces formed by type-II AGs on AGPs play a primary role in their reproductive functions. A study in apple supports the hypothesis of spatiotemporal regulation of secretion of glycoproteins in the style and ovules upon pollen tube arrival, suggesting a pivotal role for AGPs in fertilization [66, 67]. Even more interesting, AMOR, a sporophytic ovular factor derived from side chains of AGPs primes pollen tubes to respond to female gametophyte-derived attraction signals in the wishbone flower [68, 69]. Our work here on the *Hyp-GALT*s only serve to strengthen and reinforce this idea that type-II AGs on AGPs are critical to their function in reproductive tissues, most likely by serving as signaling molecules and/or nutrition sources during pollination, male gametophyte development, and fertilization.

## Conclusions

In summary, a comprehensive physiological and biochemical analysis of a set of mutants for five out of the eight known *Hyp-GALT* in two GT31 subfamilies was conducted in order to dissect their functional contributions and partial gene redundancies in different Arabidopsis organs. Despite the additive and pleiotropic effects of *GALT2*, *GALT5*, *GALT7*, *GALT8* and *GALT9* on vegetative and reproductive growth phenotypes such as rosette size, insensitivity to β-Yariv reagent, seed set and pollen development, some phenotypes exhibited more substantial regulation by specific *GALTs* and were correlated with their expression patterns. Specifically, *GALT7*, *GALT8* and *GALT9* had a dominative effect in controlling primary root growth, root tip swelling under salt stress, plant height, silique length, and pollen viability, whereas *GALT2* and *GALT5* had more pronounced effects on seed morphology, germination, and seed set. Interestingly, altered/under-glycosylated stem AGPs most likely affect the cell wall structure in the *Hyp-GALT* mutants and lead to their stunted growth. Severe effects on AGP glycosylation in various plant organs of the higher-order *Hyp-GALT* mutants were demonstrated by decreases in total Yariv-precipitated AGPs and Gal, and calcium, which in turn impact AGP function.

Finally, this study raises several questions for future studies. 1. Do these eight enzymes act on all AGPs or on a subset of AGPs? 2. What is the exact structural arrangement of the AG polysaccharides and Hyp-polysaccharide profiles in these mutants compared to WT? 3. Are there additional AGP-specific *Hyp-GALTs* that remain to be identified? 4. What is the mechanism or mode of action by which AGPs carry out their functions and precisely how are AG polysaccharides involved?

## Methods

### Plant materials and growth conditions

*A. thaliana* (Columbia-0 ecotype) was obtained from the Arabidopsis Biological Research Center (ABRC), Columbus, Ohio, USA and used as WT. The *galt2 galt5* homozygous T-DNA mutant was previously generated in our lab [30]. The *hpgt1 hpgt2 hpgt3* homozygous T-DNA mutant was obtained as a kind gift from Dr. Ogawa-Ohnishi’s lab [32]. These double and triple mutants were crossed to obtain the different combinations of higher-order *Hyp-GALT* mutants for this study. Plants were grown in soil for mutant screening, seed harvesting, and growth-stage phenotypic analysis. Plant age was counted in days after germination (DAG) whereby 1 DAG defines the first day on which green cotyledons became visible. For root harvesting, plants were grown hydroponically in germination medium and basal nutrient medium using a hydroponic growth system as described previously [70]. For phenotypic analysis of root growth, plants were grown on Murashige and Skoog (MS) medium (Caisson Laboratories, North Logan, UT, USA) containing 1% sucrose and 1 g/L Phytagel. All plants were grown under long-day conditions (16 h of light/8 h of dark, 22 °C, 60–70% relative humidity) in growth chambers at a light intensity of 122 μmol m^− 2^ s^− 1^.

### PCR genotyping

Genomic DNA was extracted from fresh young leaves, which were homogenized in a tissue disrupter using metal beads. Primer locations are indicated in Fig. 1A, and the corresponding primer sequences for genotyping are listed in Supplementary Table 2. To increase the accuracy of genotyping to isolate multiple mutants, two independent PCR reactions were used to detect mutant and WT alleles, instead of using multiplex PCR. To amplify the mutant allele, a primer set (i.e., a T-DNA primer called LBb1.3 and a gene-specific primer) was used. To amplify the WT allele, two gene-specific primers that hybridize adjacent to a T-DNA insertion site were used.

### Quantitative RT-PCR

For RNA extraction, flowers of all genotypes were harvested at 40 DAG as the expression levels were high in the inflorescence and carpel and snap-frozen in liquid nitrogen. Samples were ground to a fine powder and total RNA was extracted using Trizol reagent (Life Technologies, Grand Island, NY, USA). For real time-quantitative PCR (qRT-PCR), 1 μg of total RNA was treated with DNaseI (New England Biolabs) and used for cDNA synthesis. First-strand cDNA synthesis was performed with an oligo (dT20) primer and Superscript III reverse transcriptase (Thermo Scientific). The qPCR was set up using PerfeCT SYBR Green SuperMix (Quanta Biosciences). A total 20 μl reaction mixture contained 10 μl of PerfeCT SYBR Green SuperMix, 4 μl of 10x diluted cDNA, and 0.6 μl each of forward/ reverse primer (10 μM). Expression levels were analyzed by qRT-PCR performed in a 96-well plate on an AriaMx Real-time PCR machine (Ohio University Genomics Facility). Reaction conditions were: 95 °C for 5 min, 35 cycles of 95 °C for 10 s, 60 °C for 10 s, 72 °C for 10 s, and a final step of 72 °C for 4 min. Primer efficiency measurements and quantification cycles (Cq) were calculated using AriaMx Version 1.5 software from Agilent technologies (https://www.agilent.com/en/product/real-time-pcr-(qpcr)/real-time-pcr-(qpcr)-software/ariamx-software-download). For quantification, ACTIN gene (At3G18780) was used as reference gene and the relative gene expression data were generated using WT plants as a calibrator. Oligonucleotide sequences used for qRT-PCR are listed in Supplementary Table 3. All qRT-PCR experiments were performed in biological triplicates.

### AGP quantification by β-D-Gal-Yariv reagent

AGPs were extracted from *Hyp-GALT* mutants and WT using β-D-Gal-Yariv reagent for precipitation as described previously [71] with modifications. Briefly, rosette leaves, cauline leaves, stems, siliques were collected from 40 DAG from all genotypes whereas flowers were collected at 50 DAG for *25789* and at 40 DAG for the rest of genotypes. Exactly 0.25 g of each tissue was ground to a fine powder in presence of liquid nitrogen and mixed with 1 mL 2% CaCl_2_ followed by shaking at 200 rpm for 2–3 h at room temperature. The tissue homogenates were centrifuged at 13,000 *g* for 15 min. To 500 μl of the supernatant separated in a 1.5 mL centrifuge tubes, 200 μl of β-D-Gal-Yariv dissolved in 2% CaCl_2_ (1 mg/mL) was added. 500 μl of 2% CaCl_2_ was used as a control. After 2 h of precipitation by β-D-Gal-Yariv at room temperature, the insoluble β-Gal-Yariv-AGP complex was collected by centrifugation at 13,000 *g* for 15 min. The pellet was washed twice with 2% CaCl_2_ and then dissolved in 20 mM NaOH. The dissolved AGPs were quantified by measuring absorbance at OD_420_. Different concentrations of gum arabic (10–300 μg) (Sigma-Aldrich, St. Louis, MO, USA) dissolved in 2% CaCl_2_ were used to obtain an AGP standard curve. All measurements for different tissues were made from four biological replicates.

### Monosaccharide composition analysis by HPAEC-PAD

AGPs were extracted from siliques of 40-day-old WT and *Hyp-GALT* mutant plants. Whereas roots were harvested from hydroponically grown plants at 40 DAG. AGPs from all tissues were extracted as described previously [71], with minor modifications. For both monosaccharide composition and AGP profiling, AGPs were extracted from ~ 8–10 g of silique and root tissues which were snap-frozen with liquid nitrogen, ground to a fine powder and mixed with 2% NaCl (1 part tissue: 4 parts of 2% NaCl), followed by shaking at 200 rpm for 3 h at room temperature. Samples were centrifuged for 30 min at 13,000 *g* at room temperature. Then, 2 mL of β-D-Gal-Yariv reagent dissolved in 2% NaCl (2 mg/mL) was added to the supernatant and allowed to precipitate overnight. The precipitated AGPs were collected by centrifugation at 2000 *g* for 20 min, washed with 2% NaCl twice, resuspended in 2 mL H_2_O. Sodium dithionite was added and incubated for 15 min at 50 °C until the mixture was decolorized. The resulting solution was desalted on a PD-10 column (GE Healthcare) that had been equilibrated with water, and the eluate was freeze-dried overnight.

For monosaccharide composition analysis, approximately 500 μg of AGPs were hydrolyzed using 2 N TFA, at 121 °C for 90 min followed by removal of TFA by drying under a N_2_ (g). Samples were washed with isopropanol thrice before dissolving in 500 μl of 0.1 mM cellobiose as an internal standard. A standard sugar mixture (0.2 mM each of fucose, rhamnose, arabinose, galactose, glucose, xylose, mannose, galacturonic acid, and glucuronic acid) was employed for determining molar amounts of individual sugars by single point internal standard quantification. All samples along with standards were subjected to high-performance anion exchange chromatography with pulsed amperometric detection (HPAEC-PAD) on a Dionex ICS-500 instrument equipped with a Dionex PA-20 system (Thermo Fisher Scientific, Sunnyvale, CA, USA) essentially as described by Øbro et al., (2004) [72]. Monosaccharide compositions were calculated as averages (+/− standard error) of biological triplicates and are displayed as molar percentages.

### Calcium binding assay

For the calcium binding assay, we assayed 50 μl of AGP stocks (10 μg/mL in Mili-Q water) of WT and *Hyp-GALT* mutants’ stems, siliques, flowers and roots used for HPAEC before as described previously [73, 74]. A commercial calcium calorimetric assay kit (MAK022, Sigma-Aldrich, St. Louis, MO, USA) was used for calcium measurement as per manufacturer’s protocol.

### Germination experiment

Seeds of WT and *Hyp-GALT* mutants were sterilized, stratified at 4 °C for 3 days in the dark, and then sown on ½ MS medium and 1% sucrose agar plates. Germination percentages and rates were counted at 12 h intervals for up to 72 h after sowing, and radicle lengths were analyzed at 48 h. Approximately 36 seeds were sown for each genotype with four replicates.

### Aerial plant phenotyping

WT and *Hyp-GALT* mutants grown on soil were measured for their plant height at 30, 40, and 50 DAG and compared to WT (control) plants under normal conditions (*n* = 15) in three independent experiments. Measurement of flowering time was performed as previously described [75, 76]. Flowering time was scored as the number of DAG to the first appearance of buds at the apex and the total number of rosette leaves after the main stem has bolted 1 cm. For each replicate, flowering time was recorded from at least 15 plants per genotype in three independent experiments. Data are the averages of three replicates. Statistical significance was determined using ANOVA.

### Root and root hair growth

WT and *Hyp-GALT* mutant seeds were sterilized, stratified at 4 °C for 3 days in the dark, and then sown on ½ MS medium and 1% sucrose agar plates. Four-day-old seedlings were transferred onto ½ MS agar plates and kept in a growth chamber at 22 °C, 16 h light/ 20 °C, 8 h dark photoperiod. Primary root length of each genotype was measured from 3 d to 8 d after sowing under normal conditions (*n* = 15) in three independent experiments. Root-hair length and root-hair density at an area 3 mm to 5 mm from the root tip, 6 days after transfer on ½ MS media (10 d old-seedlings) were measured. To ensure comparable results, quantification of root hairs length was performed using 10 seedlings for of the *Hyp-GALT* mutants and WT, and 25 root hairs from each root were measured for analysis (total root hairs = 250/genotype). Root-hair density was counted for 50 seedlings in total/genotype in three independent experiments.

### Aberrant root and root hair morphology under conditional stress

For root tip analysis, seeds were germinated in ½ MS plates and grown for 3 days before transferring to MS plates supplemented with 100 mM NaCl. Root tips of plant seedlings for all genotypes grown for 10 d and visualized under a Nikon SMZ1500 stereomicroscope coupled with a CCD Infinity 2 camera. To calculate a change in root length under salt stress, root length was measured at 7 d and 14 d after transfer by capturing images using a CCD camera, which were analyzed through image analysis freeware (ImageJ; http://rsb.info.nih.gov/ij/). For monitoring root growth in response to β-Gal-Yariv reagent, WT and *Hyp-GALT* mutant seedlings were grown on ½ MS plates for 4 days before they were transferred to ½ MS plates supplemented with 50 μM β-Gal-Yariv reagent. Root length was determined on low magnification (× 10) digital images captured using a CCD camera by using image analysis freeware (ImageJ). To calculate an increase in root length in β-Gal-Yariv supplemented ½ MS media, root length was measured at 7 d and 14 d after transplanting 15 seedlings in three independent experiments.

### Seed set evaluation

The basal 15 mature siliques on the inflorescence stem at 42 DAG for 10 plants per genotype were collected from, and silique lengths were measured. For seed number, the 15 basal siliques on main inflorescence stem of 20 plants per genotype were decolorized by incubation in 70% ethanol at 37 °C overnight and were visualized under a Nikon SMZ1500 stereomicroscope coupled with a CCD Infinity 2 camera. For reciprocal cross-pollinations, 20 flowers from WT and *25789* quintuple mutants were selected at stage 12. These flowers were emasculated before pollinating them with fresh pollen obtained from flowers at stage 13. After 10 days, siliques were collected from these flowers to examine seed set.

### Scanning electron microscopy

Seeds and pollen samples were dry-mounted on aluminum stubs, covered by a 10 nm coat with palladium in a sputter coater (Anatech HUMMER 6.2 Sputtering System), and observed using a scanning electron microscope (SEM JEOL JSM-6390, HV/LV Tungsten/LaB6, Jeol USA Inc. 2012) equipped with an Energy Dispersive X-ray Spectroscopy (EDS) detector, with an accelerating voltage of 15 kV. Photographs were taken using SEM Control User Interface version 8.5 software at the Institute for Corrosion and Multiphase Technology, Ohio University. Every genotype was examined in three independent groups of roughly 20 seeds and pollen each.

### Transmission electron microscopy and toluidine blue staining

For TEM observations, the basal internodes of inflorescence stems of 6-week-old plants with the same flowering date were collected for WT and *25789* mutant. Briefly, the internodes were fixed in FAA overnight and embedded in spur resin to obtain 70 nm ultrathin sections by using an ULTRACUT N ultramicrotome (Reichert-Nissei, Tokyo, Japan) with a diamond knife and mounted on copper grids essentially as described by Suzuki et al. (2008) [63]. Specimens were viewed by FEI TEcnai G2 Spirit TEM using the Campus Microscopy and Imaging Facility at the Ohio State University.

Sections (1 μm) of resin-embedded anthers as prepared for TEM were mounted on a glass slide, stained with a toluidine blue staining solution [0.5% (w/v) toluidine blue, 0.5% (w/v) sodium borate], and the slides heated on a hot plate for 20 min. After washing out the stain, the specimen was viewed under a light microscope.

### Cytochemical staining of seeds

Seeds of all indicated genotypes were pre-hydrated in water for 1 h with shaking (200 rpm) to remove non-adherent mucilage and stained with 0.01% ruthenium red and calcofluor for 30 min each as described by [8, 40]. Ruthenium red stained seeds were examined under a Nikon SMZ1500 stereomicroscope coupled with a CCD Infinity 2 camera. For calcofluor staining, excitation was measured with 405 nm laser diode. Imaging was done using a Zeiss LSM 510 confocal microscope at Ohio University.

### Chemical analysis of adherent and non-adherent mucilage

Alterations in soluble versus adherent mucilage were assessed using the method reported by Harpaz-Saad et al. (2011) [40]. Three independent samples of 100 mg of seeds were extracted sequentially with 0.2% ammonium oxalate, 0.2 N NaOH and 2 N NaOH for 1 h each with vigorous shaking at 37 °C. Both sodium hydroxide extractions contained 3 mg/ml sodium borohydride to prevent end-degradation and were neutralized with acetic acid. Total sugar (μg/mg seed) was determined with a phenol-sulfuric assay as reported by Basu et al. (2015) [31]. Seeds used for each chemical analysis were collected from mutant and control plants cultivated together.

### In vitro pollen germination assay

Flowers collected from WT and *Hyp-GALT* mutant plants 1 to 2 weeks after bolting were used for the examination of pollen tube phenotypes. Individual open flowers were germinated in vitro as described previously [77, 78], on solid germination medium (0.01% H_3_BO_3_, 1 mM Ca(NO_3_)_2_, 1 mM KCl, 1 mM CaCl_2_, 10% sucrose, 0.03% casein enzymatic hydrolysate, 0.01% myo-inositol, 0.1 mM spermidine, 10 mM GABA, 500 μM methyl jasmonate and 1.0% low-melting agarose, pH 7.5 and 30 μM β-Gal-Yariv reagent) at 22 °C and 100% humidity in the dark. Pollen tube germination rates were calculated by dividing the total number of germinated tubes by the number of grains. Images and measurements of pollen tubes were done at 10× magnification in a Nikon Phot-lab2 microscope coupled with a SPOT RT color CCD camera and SPOT 4.2 analysis software.

## Supplementary Information

**Additional file 1: Supplemental Figure 1.** In silico gene expression profiles of the eight *Hyp-GALT* genes in Arabidopsis organs/ tissues. Araport (Cheng et al., 2016) utilizes 113 public RNA-seq data sets along with annotation contributions from NCBI, UniProt, and labs conducting *Arabidopsis thaliana* research to obtain gene expression values based on transcript abundance normalized in accordance with a reference gene in the experiment. **Supplemental Figure 2**. Gene expression analysis of the eight *Hyp-GALT* genes during seed development. BAR eFP browser (Lee et al., 2010) displays gene expression profiles based on laser-capture micro-dissected seeds during various stages of seed development. **Supplemental Figure 3.** Growth phenotype of WT and higher-order *Hyp-GALT* mutants on soil. **A.** WT and mutant seedlings were sown on ½ MS media and transferred to soil at 10 DAG; photos were taken over a period of four weeks on soil. Rosette sizes of *789, 2578, 2579* and *25789* were smaller than wild type throughout the 4 weeks. **B.** Total number of rosette leaves were counted for mutants and WT plants after the main stem bolted 1 cm. Data for number of rosette leaves are means±SE of measurements from three independent experiments (total *n* = 50). An analysis of variance (ANOVA) on these mutants yielded no significant variation among conditions. DAG, days after germination. **Supplemental Figure 4**. Quantification of root hair length and root hair density of wild type and higher-order *Hyp-GALT* mutants on ½ MS media at 7 DAG. Data for root hair length are means ± SE of measurements from three independent experiments (*n* = 80). Data for root hair density are means ± SE of measurements from three independent experiments (total *n* = 50). An analysis of variance (ANOVA) on these mutants yielded significant variation among conditions. A post hoc Tukey test was applied to see which groups were significantly different from wild type (Col-0). Asterisks indicate significantly reduced root hair length and density compared with WT. *P < .05, **P < 0.005, ***P < 0.001. DAG, days after germination. **Supplemental Figure 5**. Salt induced inhibition of vertically grown primary roots of WT and higher-order *Hyp-GALT* mutants on ½ MS supplemented with 100 mM NaCl. **A.** Seedlings of WT and mutants were grown vertically on ½ MS media for 4 DAG and pictures were taken 7 days after transfer to ½ MS supplemented with 100 mM NaCl. Scale bar = 1 cm. **B.** 10 days after transfer to ½ MS supplemented with 100 mM NaCl. Scale bar = 1.0 mm. **C.** Root elongation (i.e. increase in root length of 4-day-old seedlings after transfer onto ½ MS supplemented with 100 mM NaCl) was measured after 7, 14 and 21 days. Data are means±SE of measurements from three independent experiments (total *n* = 40). An analysis of variance (ANOVA) on these mutants yielded significant variation among conditions. A post hoc Tukey test was applied to see which groups were significantly different from WT. *P < .05, **P < 0.005, ***P < 0.001. DAG, days after germination. **Supplemental Figure 6.** Mean seed set of the basal 15 siliques on the main inflorescence of the *Hyp-GALT* mutants compared to WT. The fifteen siliques (starting from the base) were used to quantify seed set in 45-days-old plants grown on soil. Data presented are mean ± SD (*n* = 20 plants in three independent experiments). **Supplemental Figure 7.** Mean silique lengths of the basal 15 siliques on the main inflorescence of the *Hyp-GALT* mutants compared to WT. The fifteen siliques (starting from the base were used to quantify silique length in 45-day-old plants grown on soil. Data presented are mean±SD (*n*=10 plants from two independent experiments). **Supplemental Figure 8.** Higher-order *Hyp-GALT* mutant flower morphology compared to WT flower morphology. The *25789* mutant had smaller fertile flowers compared to WT, but the floral organization was similar (*n*=10 flowers from 5 plants each). Scale bar=1.0 mm. **Supplementary Table 1**. Information on the Hyp-GALT enzymes, genes, and their genetic mutants. The two independent *GALT2-6* T-DNA lines have been characterised and they had same phenotypes as reported by Basu et al. (2015) [30] and Basu et al. (2015) [31]. The *GALT7-9* T-DNA insertion lines were identified and confirmed by Ogawa-Ohnishi and Matsubayashi (2015) [32]. Also, the subcellular localization of GALT2-9 mentioned here were reported in these studies. **Supplementary Table 2**. Oligonucleotides used for genotyping of the *Hyp-GALT* mutants. **Supplementary Table 3.** Oligonucleotide sequences used for qRT-PCR of the *Hyp-GALT* mutants. **Supplementary Table 4.** The *Hyp-GALT* mutants display reduced total sugars (μg/mg seed ± SE) compared to WT in Arabidopsis seed mucilage.

## Data Availability

The datasets used and/or analyzed during the current study are available from the corresponding author upon reasonable request.

## Abbreviations

AGP: Arabinogalactan-protein
HRGP: Hydroxyproline-rich glycoprotein
TEM: Transmission electron microscopy
HPAEC-PAD: High-Performance Anion-Exchange Chromatography with Pulsed Amperometric Detection
GlcA: Glucuronic acid
AG: Arabinogalactan
GPI: Glycosylphosphatidylinositol
Hyp-*GALT*: Hydroxyproline-galactosyltransferase
Gal: Galactose
CAZy: Carbohydrate active enzyme (database)
GT: Glycosyltransferase
WT: Wild-type
ACC: Amino-cyclopropane-carboxylate
DAG: Days after germination

## References

[1] Hervé C, Siméon A, Jam M, Cassin A, Johnson KL, Salmeán AA (2016). Arabinogalactan proteins have deep roots in eukaryotes: identification of genes and epitopes in brown algae and their role in *Fucus serratus* embryo development. New Phytol.

[2] Palacio-López K, Tinaz B, Holzinger A, Domozych DS. Arabinogalactan proteins and the extracellular matrix of Charophytes: a sticky business. Front. Plant Sci. 2019;10. 10.3389/fpls.2019.00447. 10.3389/fpls.2019.00447 PMC6474363 31031785

[3] Schultz C, Johnson K, Currie G, Bacic A (2000). The classical arabinogalactan protein gene family of Arabidopsis. Plant Cell.

[4] Showalter AM, Keppler B, Lichtenberg J, Gu D, Welch LR (2010). A bioinformatics approach to the identification, classification, and analysis of hydroxyproline-rich glycoproteins. Plant Physiol.

[5] Schultz CJ, Ferguson KL, Lahnstein J, Bacic A (2004). Post-translational modifications of arabinogalactan-peptides of *Arabidopsis thaliana* endoplasmic reticulum and glycosylphosphatidylinositol-anchor signal cleavage sites and hydroxylation of proline. J Biol Chem.

[6] Seifert GJ, Roberts K (2007). The biology of arabinogalactan proteins. Ann Rev Plant Biol.

[7] Majewska-Sawka A, Nothnagel EA (2000). The multiple roles of arabinogalactan proteins in plant development. Plant Physiol.

[8] Willats WGT, Knox JP (1996). A role for arabinogalactan-proteins in plant cell expansion: evidence from studies on the interaction of β-glucosyl Yariv reagent with seedlings of *Arabidopsis thaliana*. Plant J.

[9] Coimbra S, Costa M, Jones B, Mendes MA, Pereira LG (2009). Pollen grain development is compromised in Arabidopsis *agp6 agp11* null mutants. J Exp Bot.

[10] Ellis M, Egelund J, Schultz CJ, Bacic A (2010). Arabinogalactan-proteins: key regulators at the cell surface?. Plant Physiol.

[11] Zhang Y, Yang J, Showalter AM (2011). AtAGP18, a lysine-rich arabinogalactan protein in Arabidopsis thaliana, functions in plant growth and development as a putative co-receptor for signal transduction. Plant Signal Behav.

[12] Nguema-Ona E, Coimbra S, Vicre-Gibouin M, Mollet J-C, Driouich A (2012). Arabinogalactan proteins in root and pollen-tube cells: distribution and functional aspects. Ann Bot.

[13] Olmos E, García De La Garma J, Gomez-Jimenez MC, Fernandez-Garcia N. Arabinogalactan proteins are involved in salt-adaptation and vesicle trafficking in tobacco BY-2 cell cultures. Front Plant Sci. 2017;8. 10.3389/fpls.2017.01092. 10.3389/fpls.2017.01092 PMC5476920 28676820

[14] Kieliszewski MJ, O’Neill M, Leykam J, Orlando R (1995). Tandem mass spectrometry and structural elucidation of glycopeptides from a hydroxyproline-rich plant cell wall glycoprotein indicate that contiguous hydroxyproline residues are the major sites of hydroxyproline *O*-arabinosylation. J Biol Chem.

[15] Tan L, Qiu F, Lamport DTA, Kieliszewski MJ (2004). Structure of a hydroxyproline (Hyp)-arabinogalactan polysaccharide from repetitive ala-Hyp expressed in transgenic *Nicotiana tabacum*. J Biol Chem.

[16] Kieliszewski MJ, Kamyab A, Leykam JF, Lamport DTA (1992). A histidine-rich extensin from *Zea mays* is an arabinogalactan protein. Plant Physiol.

[17] Du H, Clarke AE, Bacic A (1996). Arabinogalactan-proteins: a class of extracellular matrix proteoglycans involved in plant growth and development. Trends Cell Biol.

[18] Nothnagel EA. Proteoglycans and related components in plant cells. International review of cytology: Elsevier; 1997. p. 195–291. 10.1016/S0074-7696(08)62118-X. 10.1016/s0074-7696(08)62118-x 9161008

[19] Qu Y, Egelund J, Gilson PR, Houghton F, Gleeson PA, Schultz CJ (2008). Identification of a novel group of putative *Arabidopsis thaliana* β-(1,3)-galactosyltransferases. Plant Mol Biol.

[20] Suzuki T, Narciso JO, Zeng W, van de Meene A, Yasutomi M, Takemura S (2017). KNS4/UPEX1: a type II arabinogalactan *β* -(1,3)-galactosyltransferase required for pollen exine development. Plant Physiol.

[21] Geshi N, Johansen JN, Dilokpimol A, Rolland A, Belcram K, Verger S (2013). A galactosyltransferase acting on arabinogalactan protein glycans is essential for embryo development in Arabidopsis. Plant J.

[22] Dilokpimol A, Poulsen CP, Vereb G, Kaneko S, Schulz A, Geshi N (2014). Galactosyltransferases from *Arabidopsis thaliana* in the biosynthesis of type II arabinogalactan: molecular interaction enhances enzyme activity. BMC Plant Biol.

[23] Knoch E, Dilokpimol A, Tryfona T, Poulsen CP, Xiong G, Harholt J (2013). A β–glucuronosyltransferase from *Arabidopsis thaliana* involved in biosynthesis of type II arabinogalactan has a role in cell elongation during seedling growth. Plant J.

[24] Dilokpimol A, Geshi N (2014). *Arabidopsis thaliana* glucuronosyltransferase in family GT14. Plant Signal Behav.

[25] Wu Y, Williams M, Bernard S, Driouich A, Showalter AM, Faik A (2010). Functional identification of two nonredundant *Arabidopsis* α (1,2) fucosyltransferases specific to arabinogalactan proteins. J Biol Chem.

[26] Liang Y, Basu D, Pattathil S, Xu W, Venetos A, Martin SL (2013). Biochemical and physiological characterization of *fut4* and *fut6* mutants defective in arabinogalactan-protein fucosylation in Arabidopsis. J Exp Bot.

[27] Tryfona T, Theys TE, Wagner T, Stott K, Keegstra K, Dupree P (2014). Characterisation of FUT4 and FUT6 α-(1→2)-fucosyltransferases reveals that absence of root arabinogalactan fucosylation increases Arabidopsis root growth salt sensitivity. Muday G, editor. PLoS One.

[28] Gille S, Sharma V, Baidoo EEK, Keasling JD, Scheller HV, Pauly M (2013). Arabinosylation of a yariv-precipitable cell wall polymer impacts plant growth as exemplified by the Arabidopsis glycosyltransferase mutant ray1. Mol Plant.

[29] Narciso JO, Zeng W, Ford K, Lampugnani ER, Humphries J, Austarheim I (2021). Biochemical and functional characterization of GALT8, an Arabidopsis GT31 β-(1,3)-galactosyltransferase that influences seedling development. Front Plant Sci.

[30] Basu D, Wang W, Ma S, DeBrosse T, Poirier E, Emch K (2015). Two hydroxyproline galactosyltransferases, GALT5 and GALT2, function in arabinogalactan-protein glycosylation, growth and development in Arabidopsis. PLOS ONE.

[31] Basu D, Tian L, Wang W, Bobbs S, Herock H, Travers A, et al. A small multigene hydroxyproline-*O*-galactosyltransferase family functions in arabinogalactan-protein glycosylation, growth and development in Arabidopsis. BMC Plant Biol. 2015;15. 10.1186/s12870-015-0670-7. 10.1186/s12870-015-0670-7 PMC4687291 26690932

[32] Ogawa-Ohnishi M, Matsubayashi Y (2015). Identification of three potent hydroxyproline *O* -galactosyltransferases in Arabidopsis. Plant J.

[33] Showalter AM, Basu D (2016). Glycosylation of arabinogalactan-proteins essential for development in Arabidopsis. Commun Integr Biol.

[34] Basu D, Tian L, Debrosse T, Poirier E, Emch K, Herock H, et al. Glycosylation of a fasciclin-like arabinogalactan-protein (SOS5) mediates root growth and seed mucilage adherence via a cell wall receptor-like kinase (FEI1/FEI2) pathway in Arabidopsis. PLoS One. 2016;11. 10.1371/journal.pone.0145092. 10.1371/journal.pone.0145092 PMC4701510 26731606

[35] Zhang Y, Held MA, Kaur D, Showalter AM (2021). CRISPR-Cas9 multiplex genome editing of the hydroxyproline-*O*-galactosyltransferase gene family alters arabinogalactan-protein glycosylation and function in Arabidopsis. BMC Plant Biol.

[36] Le BH, Cheng C, Bui AQ, Wagmaister JA, Henry KF, Pelletier J (2010). Global analysis of gene activity during Arabidopsis seed development and identification of seed-specific transcription factors. Proc Natl Acad Sci U S A.

[37] Nordborg M, Borevitz JO, Bergelson J, Berry CC, Chory J, Hagenblad J (2002). The extent of linkage disequilibrium in *Arabidopsis thaliana*. Nat Genet.

[38] Kitazawa K, Tryfona T, Yoshimi Y, Hayashi Y, Kawauchi S, Antonov L (2013). β-Galactosyl yariv reagent binds to the β-1,3-galactan of arabinogalactan proteins. Plant Physiol.

[39] Ding L, Zhu J-K (1997). A role for arabinogalactan-proteins in root epidermal cell expansion. Planta..

[40] Harpaz-Saad S, McFarlane HE, Xu S, Divi UK, Forward B, Western TL (2011). Cellulose synthesis via the FEI2 RLK/SOS5 pathway and CELLULOSE SYNTHASE 5 is required for the structure of seed coat mucilage in Arabidopsis. The Plant J..

[41] Strasser R, Bondili JS, Vavra U, Schoberer J, Svoboda B, Glössl J (2007). A unique β1,3-galactosyltransferase is indispensable for the biosynthesis of N-glycans containing Lewis a structures in *Arabidopsis thaliana*. Plant Cell.

[42] Lamport DTA, Kieliszewski MJ, Showalter AM (2006). Salt stress upregulates periplasmic arabinogalactan proteins: using salt stress to analyse AGP function. New Phytol.

[43] Lamport DTA, Varnai P, Seal CE (2014). Back to the future with the AGP–Ca2+ flux capacitor. Ann Bot.

[44] Duan J, Zheng Y, Dong Q, Fang J (2004). Structural analysis of a pectic polysaccharide from the leaves of Diospyros kaki. Phytochemistry..

[45] Tan L, Eberhard S, Pattathil S, Warder C, Glushka J, Yuan C (2013). An Arabidopsis cell wall proteoglycan consists of pectin and Arabinoxylan covalently linked to an arabinogalactan protein. Plant Cell.

[46] Tan L, Showalter AM, Egelund J, Hernandez-Sanchez A, Doblin MS, Bacic A. Arabinogalactan-proteins and the research challenges for these enigmatic plant cell surface proteoglycans. Front Plant Sci. 2012;3. 10.3389/fpls.2012.00140. 10.3389/fpls.2012.00140 PMC3384089 22754559

[47] Lopez-Hernandez F, Tryfona T, Rizza A, Yu XL, Harris MOB, Webb AAR (2020). Calcium binding by arabinogalactan polysaccharides is important for normal plant development. Plant Cell.

[48] Gaspar Y, Johnson KL, McKenna JA, Bacic A, Schultz CJ (2001). The complex structures of arabinogalactan-proteins and the journey towards understanding function. Plant Mol Biol.

[49] Johnson KL, Jones BJ, Bacic A, Schultz CJ (2003). The Fasciclin-like arabinogalactan proteins of arabidopsis. A multigene family of putative cell adhesion molecules. Plant Physiol.

[50] Yang J, Sardar HS, McGovern KR, Zhang Y, Showalter AM (2007). A lysine-rich arabinogalactan protein in Arabidopsis is essential for plant growth and development, including cell division and expansion. Plant J..

[51] Velasquez SM, Ricardi MM, Dorosz JG, Fernandez PV, Nadra AD, Pol-Fachin L (2011). *O*-glycosylated cell wall proteins are essential in root hair growth. Science..

[52] Velasquez SM, Ricardi MM, Poulsen CP, Oikawa A, Dilokpimol A, Halim A (2015). Complex regulation of prolyl-4-hydroxylases impacts root hair expansion. Mol Plant.

[53] Hengel AJV, Roberts K (2003). AtAGP30, an arabinogalactan-protein in the cell walls of the primary root, plays a role in root regeneration and seed germination. Plant J.

[54] Liu C, Mehdy MC (2007). A nonclassical arabinogalactan protein gene highly expressed in vascular tissues, AGP31, is transcriptionally repressed by methyl Jasmonic acid in Arabidopsis. Plant Physiol.

[55] MacMillan CP, Mansfield SD, Stachurski ZH, Evans R, Southerton SG (2010). Fasciclin-like arabinogalactan proteins: specialization for stem biomechanics and cell wall architecture in *Arabidopsis* and *Eucalyptus*. The Plant J..

[56] Liu E, MacMillan CP, Shafee T, Ma Y, Ratcliffe J, van de Meene A, et al. Fasciclin-like arabinogalactan-protein 16 (FLA16) is required for stem development in *Arabidopsis*. Front Plant Sci. 2020;11. 10.3389/fpls.2020.615392. 10.3389/fpls.2020.615392 PMC7758453 33362841

[57] Liu S, Tang Y, Ruan N, Dang Z, Huang Y, Miao W (2020). The rice BZ1 locus is required for glycosylation of arabinogalactan proteins and galactolipid and plays a role in both mechanical strength and leaf color. Rice..

[58] Sardar HS, Yang J, Showalter AM (2006). Molecular interactions of arabinogalactan proteins with cortical microtubules and F-actin in bright yellow-2 tobacco cultured cells. Plant Physiol.

[59] Nguema-Ona E, Bannigan A, Chevalier L, Baskin TI, Driouich A (2007). Disruption of arabinogalactan proteins disorganizes cortical microtubules in the root of *Arabidopsis thaliana*. Plant J..

[60] Driouich A, Baskin TI (2008). Intercourse between cell wall and cytoplasm exemplified by arabinogalactan proteins and cortical microtubules. Am J Bot.

[61] Goldberg RB, de Paiva G, Yadegari R (1994). Plant embryogenesis: zygote to seed. Science..

[62] Voiniciuc C, Zimmermann E, Schmidt MH-W, Günl M, Fu L, North HM, et al. Extensive natural variation in *Arabidopsis* seed mucilage structure. Front. Plant Sci. 2016;7. 10.3389/fpls.2016.00803. 10.3389/fpls.2016.00803 PMC4894908 27375657

[63] Suzuki T, Masaoka K, Nishi M, Nakamura K, Ishiguro S (2008). Identification of *kaonashi* mutants showing abnormal pollen exine structure in *Arabidopsis thaliana*. Plant Cell Physiol.

[64] Costa M, Nobre MS, Becker JD, Masiero S, Amorim MI, Pereira LG (2013). Expression-based and co-localization detection of arabinogalactan protein 6 and arabinogalactan protein 11 interactors in *Arabidopsis* pollen and pollen tubes. BMC Plant Biol.

[65] Leszczuk A, Kozioł A, Szczuka E, Zdunek A (2019). Analysis of AGP contribution to the dynamic assembly and mechanical properties of cell wall during pollen tube growth. Plant Sci.

[66] Losada JM, Herrero M (2012). Arabinogalactan-protein secretion is associated with the acquisition of stigmatic receptivity in the apple flower. Ann Bot.

[67] Losada JM, Herrero M. Pollen tube access to the ovule is mediated by glycoprotein secretion on the obturator of apple (*Malus × domestica* , Borkh). Ann Bot. 2017:mcw276. 10.1093/aob/mcw276. 10.1093/aob/mcw276 PMC5604596 28137704

[68] Dresselhaus T, Coimbra S (2016). Plant reproduction: AMOR enables males to respond to female signals. Curr Biol.

[69] Jiao J, Mizukami AG, Sankaranarayanan S, Yamguchi J, Itami K, Higashiyama T (2017). Structure-activity relation of AMOR sugar molecule that activates pollen-tubes for ovular guidance. Plant Physiol.

[70] Conn SJ, Hocking B, Dayod M, Xu B, Athman A, Henderson S (2013). Protocol: Optimising hydroponic growth systems for nutritional and physiological analysis of *Arabidopsis thaliana* and other plants. Plant Methods.

[71] Lamport D. Preparation of arabinogalactan glycoproteins from plant tissue. Bio-Protocol. 2013;3. 10.21769/BioProtoc.918.

[72] Øbro J, Harholt J, Scheller HV, Orfila C (2004). Rhamnogalacturonan I in *Solanum tuberosum* tubers contains complex arabinogalactan structures. Phytochem..

[73] Gindler EM, King JD (1972). Rapid colorimetric determination of calcium in biologic fluids with methylthymol blue. Am J Clin Pathol.

[74] Zhang Y, Held MA, Showalter AM (2020). Elucidating the roles of three β-glucuronosyltransferases (GLCATs) acting on arabinogalactan-proteins using a CRISPR-Cas9 multiplexing approach in Arabidopsis. BMC Plant Biol.

[75] Chen R, Jiang H, Li L, Zhai Q, Qi L, Zhou W (2012). The Arabidopsis mediator subunit med25 differentially regulates jasmonate and abscisic acid signaling through interacting with the MYC2 and ABI5 transcription factors. Plant Cell.

[76] Zhai Q, Zhang X, Wu F, Feng H, Deng L, Xu L (2015). Transcriptional mechanism of jasmonate receptor COI1-mediated delay of flowering time in *Arabidopsis*. Plant Cell.

[77] Rodriguez-Enriquez MJ, Mehdi S, Dickinson HG, Grant-Downton RT (2013). A novel method for efficient in vitro germination and tube growth of *Arabidopsis thaliana* pollen. New Phytol.

[78] MacAlister CA, Ortiz-Ramírez C, Becker JD, Feijó JA, Lippman ZB (2016). Hydroxyproline *O*-arabinosyltransferase mutants oppositely alter tip growth in *Arabidopsis thaliana* and *Physcomitrella patens*. Plant J.

