# Supplementary material for: Functional characterization of hydroxyproline-O-galactosyltransferases for Arabidopsis arabinogalactan-protein synthesis
Source: BMC Plant Biol. 2021 Dec 13;21:590. doi: 10.1186/s12870-021-03362-2 (PMC8667403; doi:10.1186/s12870-021-03362-2)
Supplement: Supplementary file 1 — Additional file 1: Supplemental Figure 1. In silico gene expression profiles of the eight Hyp-GALT genes in Arabidopsis organs/ tissues. Araport (Cheng et al., 2016) utilizes 113 public RNA-seq data sets along with annotation contributions from NCBI, UniProt, and labs conducting Arabidopsis thaliana research to obtain gene expression values based on transcript abundance normalized in accordance with a reference gene in the experiment. Supplemental Figure 2. Gene expression analysis of the eight Hyp-GALT genes during seed development. BAR eFP browser (Lee et al., 2010) displays gene expression profiles based on laser-capture micro-dissected seeds during various stages of seed development. Supplemental Figure 3. Growth phenotype of WT and higher-order Hyp-GALT mutants on soil. A. WT and mutant seedlings were sown on ½ MS media and transferred to soil at 10 DAG; photos were taken over a period of four weeks on soil. Rosette sizes of 789, 2578, 2579 and 25789 were smaller than wild type throughout the 4 weeks. B. Total number of rosette leaves were counted for mutants and WT plants after the main stem bolted 1 cm. Data for number of rosette leaves are means±SE of measurements from three independent experiments (total n = 50). An analysis of variance (ANOVA) on these mutants yielded no significant variation among conditions. DAG, days after germination. Supplemental Figure 4. Quantification of root hair length and root hair density of wild type and higher-order Hyp-GALT mutants on ½ MS media at 7 DAG. Data for root hair length are means ± SE of measurements from three independent experiments (n = 80). Data for root hair density are means ± SE of measurements from three independent experiments (total n = 50). An analysis of variance (ANOVA) on these mutants yielded significant variation among conditions. A post hoc Tukey test was applied to see which groups were significantly different from wild type (Col-0). Asterisks indicate significantly reduced root hair length and d [file 12870_2021_3362_MOESM1_ESM.pptx]

## Slide 1
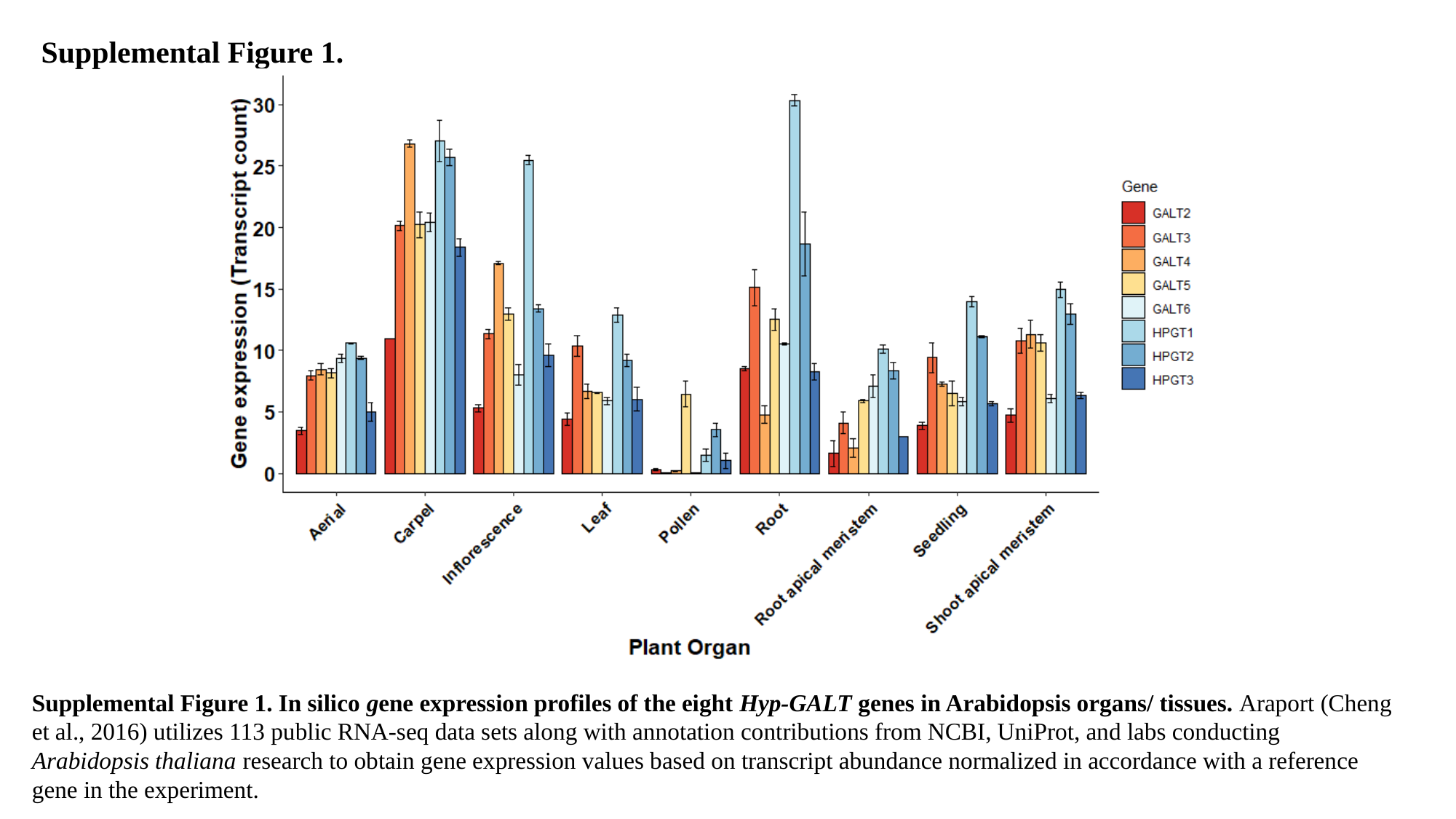

Supplemental Figure 1.
Supplemental Figure 1. In silico gene expression profiles of the eight Hyp-GALT genes in Arabidopsis organs/ tissues. Araport (Cheng et al., 2016) utilizes 113 public RNA-seq data sets along with annotation contributions from NCBI, UniProt, and labs conducting Arabidopsis thaliana research to obtain gene expression values based on transcript abundance normalized in accordance with a reference gene in the experiment.

## Slide 2
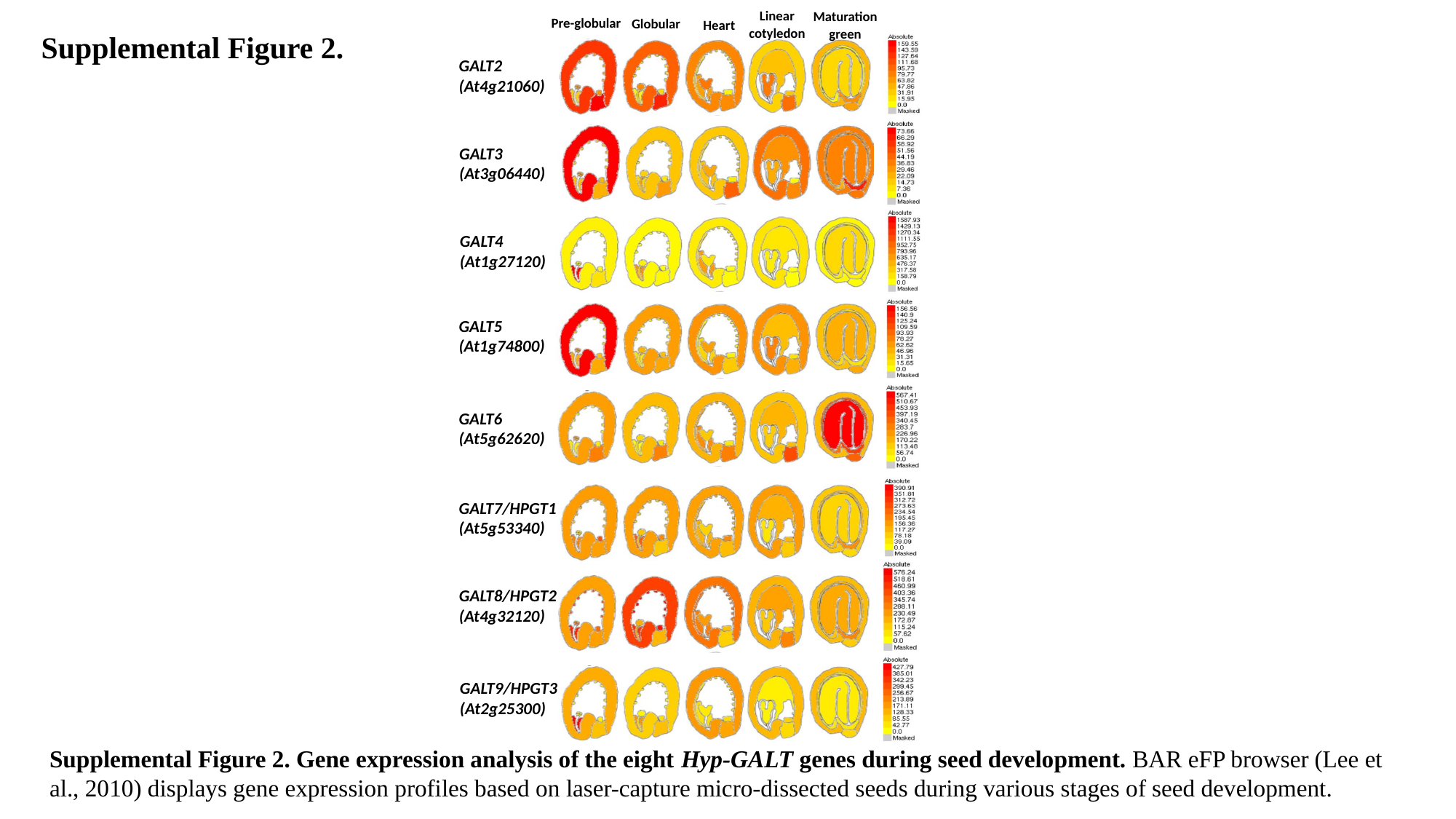

Linear cotyledon
Maturation green
Pre-globular
Globular
Heart
GALT2
(At4g21060)
GALT3
(At3g06440)
GALT4
(At1g27120)
GALT5
(At1g74800)
GALT6
(At5g62620)
GALT7/HPGT1
(At5g53340)
GALT8/HPGT2
(At4g32120)
GALT9/HPGT3
(At2g25300)
Supplemental Figure 2.
Supplemental Figure 2. Gene expression analysis of the eight Hyp-GALT genes during seed development. BAR eFP browser (Lee et al., 2010) displays gene expression profiles based on laser-capture micro-dissected seeds during various stages of seed development.

## Slide 3
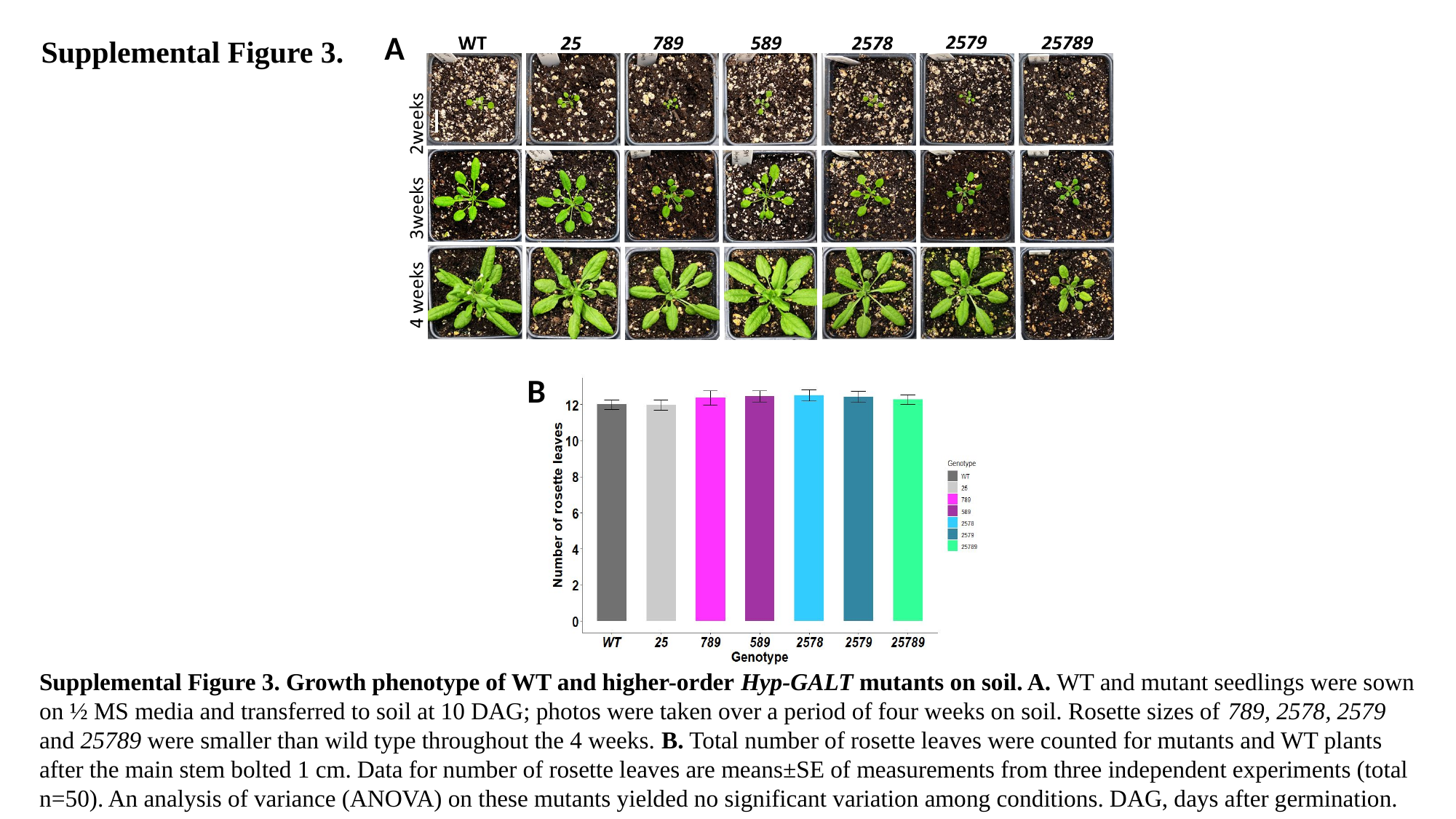

A
B
Supplemental Figure 3.
Supplemental Figure 3. Growth phenotype of WT and higher-order Hyp-GALT mutants on soil. A. WT and mutant seedlings were sown on ½ MS media and transferred to soil at 10 DAG; photos were taken over a period of four weeks on soil. Rosette sizes of 789, 2578, 2579 and 25789 were smaller than wild type throughout the 4 weeks. B. Total number of rosette leaves were counted for mutants and WT plants after the main stem bolted 1 cm. Data for number of rosette leaves are means±SE of measurements from three independent experiments (total n=50). An analysis of variance (ANOVA) on these mutants yielded no significant variation among conditions. DAG, days after germination.

## Slide 4
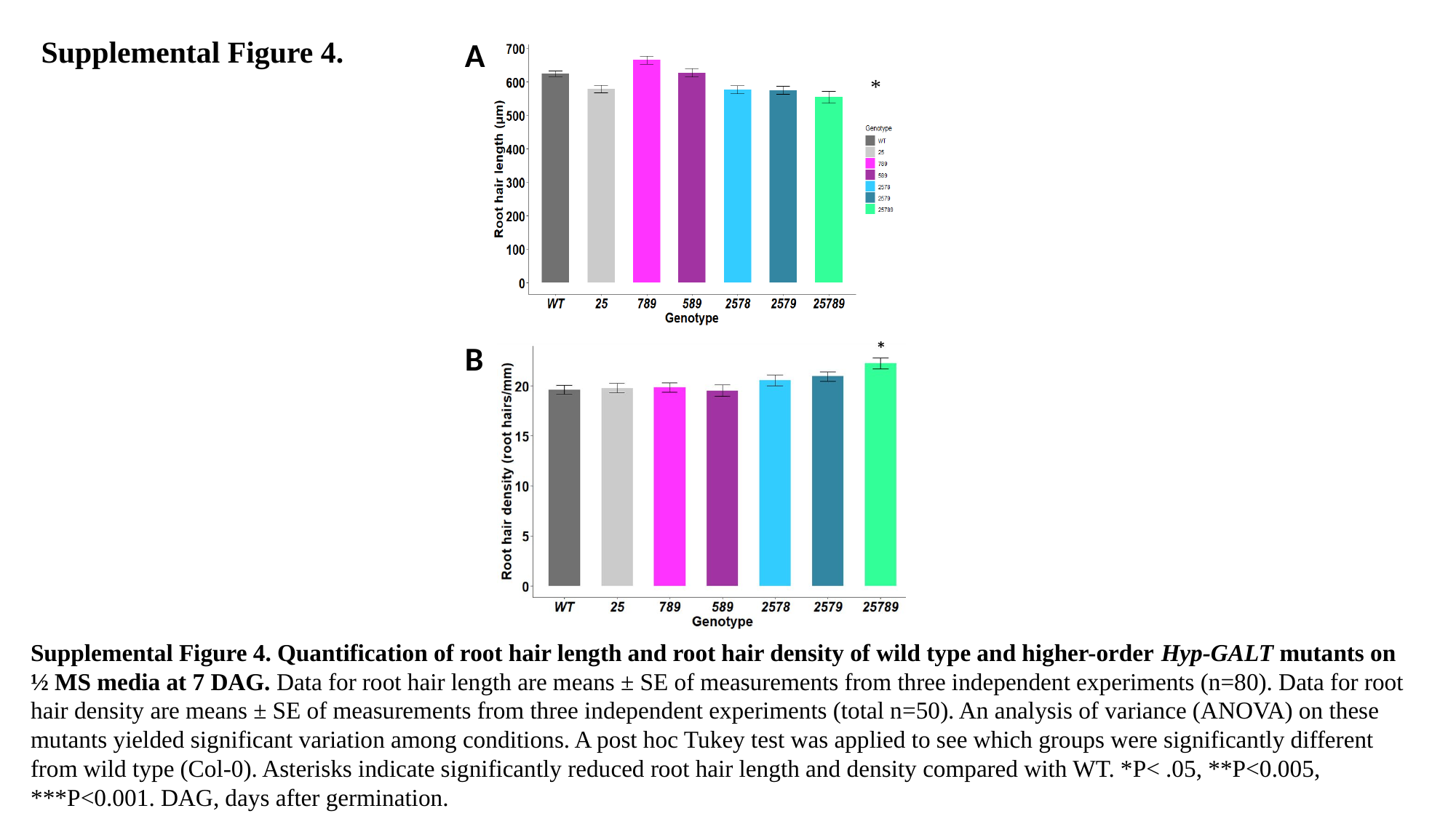

Supplemental Figure 4.
A
*
B
Supplemental Figure 4. Quantification of root hair length and root hair density of wild type and higher-order Hyp-GALT mutants on ½ MS media at 7 DAG. Data for root hair length are means ± SE of measurements from three independent experiments (n=80). Data for root hair density are means ± SE of measurements from three independent experiments (total n=50). An analysis of variance (ANOVA) on these mutants yielded significant variation among conditions. A post hoc Tukey test was applied to see which groups were significantly different from wild type (Col-0). Asterisks indicate significantly reduced root hair length and density compared with WT. *P< .05, **P<0.005, ***P<0.001. DAG, days after germination.

## Slide 5
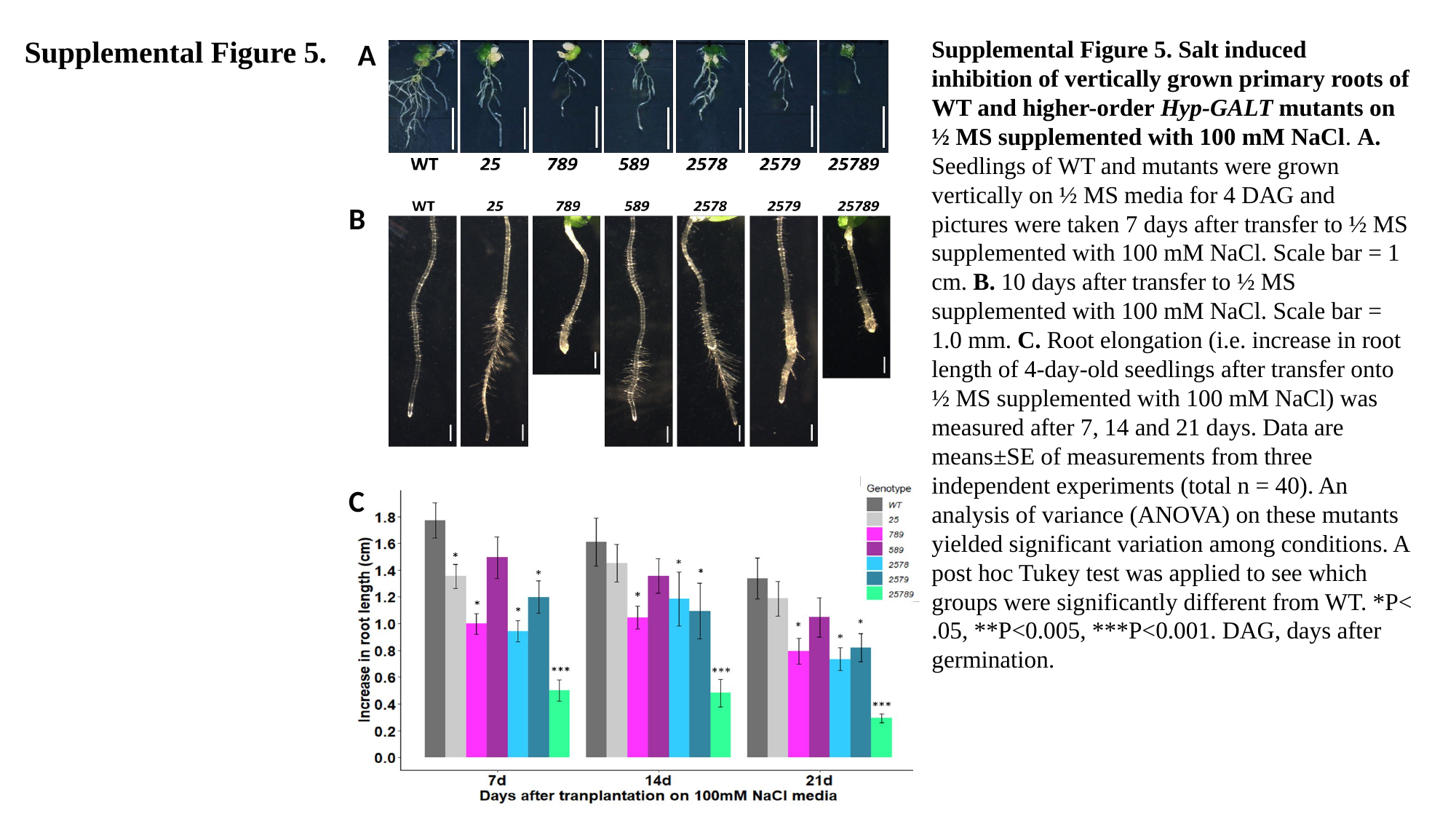

Supplemental Figure 5.
Supplemental Figure 5. Salt induced inhibition of vertically grown primary roots of WT and higher-order Hyp-GALT mutants on ½ MS supplemented with 100 mM NaCl. A. Seedlings of WT and mutants were grown vertically on ½ MS media for 4 DAG and pictures were taken 7 days after transfer to ½ MS supplemented with 100 mM NaCl. Scale bar = 1 cm. B. 10 days after transfer to ½ MS supplemented with 100 mM NaCl. Scale bar = 1.0 mm. C. Root elongation (i.e. increase in root length of 4-day-old seedlings after transfer onto ½ MS supplemented with 100 mM NaCl) was measured after 7, 14 and 21 days. Data are means±SE of measurements from three independent experiments (total n = 40). An analysis of variance (ANOVA) on these mutants yielded significant variation among conditions. A post hoc Tukey test was applied to see which groups were significantly different from WT. *P< .05, **P<0.005, ***P<0.001. DAG, days after germination.
A
B
C

## Slide 6
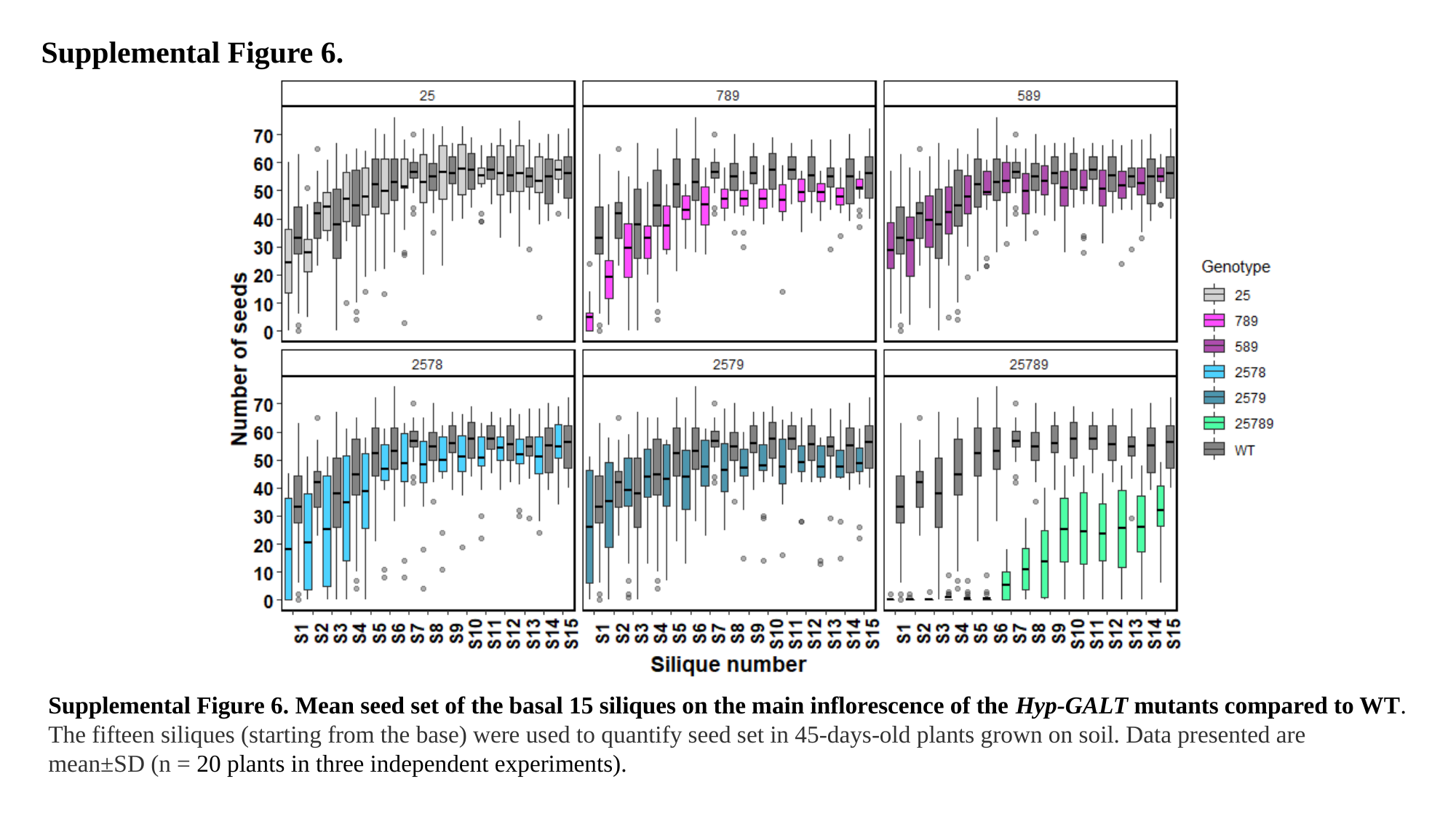

Supplemental Figure 6.
Supplemental Figure 6. Mean seed set of the basal 15 siliques on the main inflorescence of the Hyp-GALT mutants compared to WT. The fifteen siliques (starting from the base) were used to quantify seed set in 45-days-old plants grown on soil. Data presented are mean±SD (n = 20 plants in three independent experiments).

## Slide 7
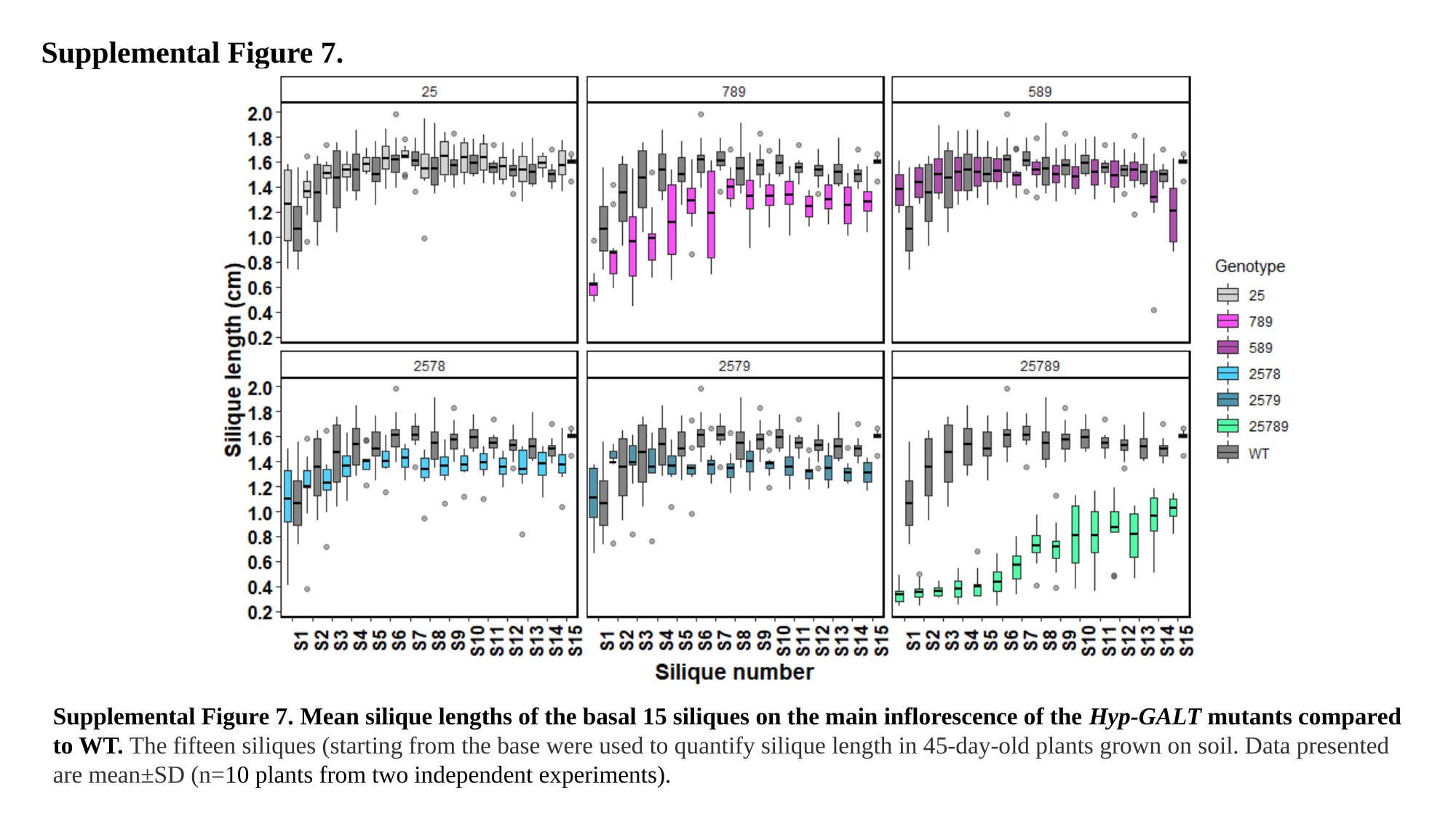

Supplemental Figure 7.
Supplemental Figure 7. Mean silique lengths of the basal 15 siliques on the main inflorescence of the Hyp-GALT mutants compared to WT. The fifteen siliques (starting from the base were used to quantify silique length in 45-day-old plants grown on soil. Data presented are mean±SD (n=10 plants from two independent experiments).

## Slide 8
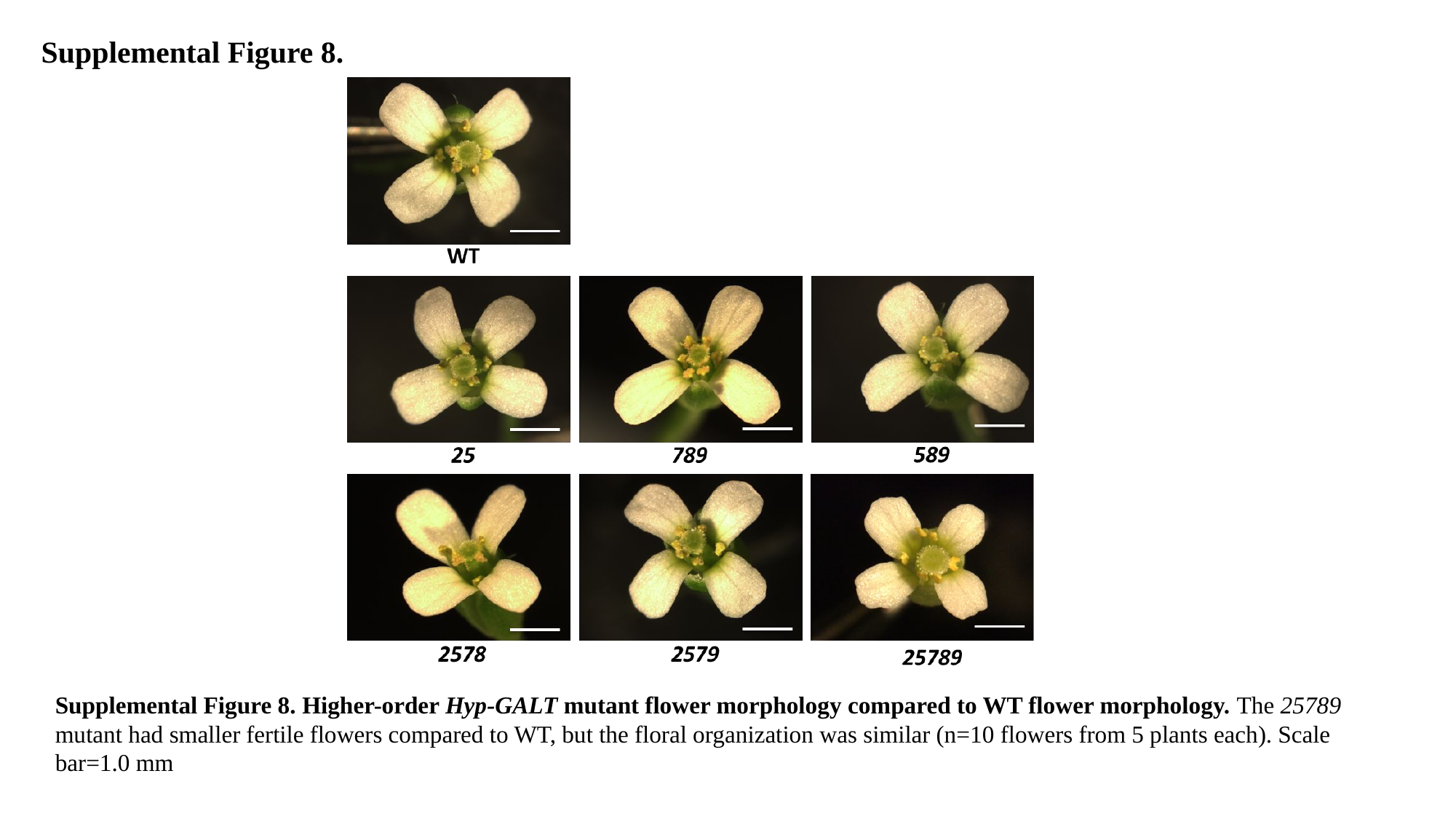

Supplemental Figure 8.
Supplemental Figure 8. Higher-order Hyp-GALT mutant flower morphology compared to WT flower morphology. The 25789 mutant had smaller fertile flowers compared to WT, but the floral organization was similar (n=10 flowers from 5 plants each). Scale bar=1.0 mm

## Slide 9
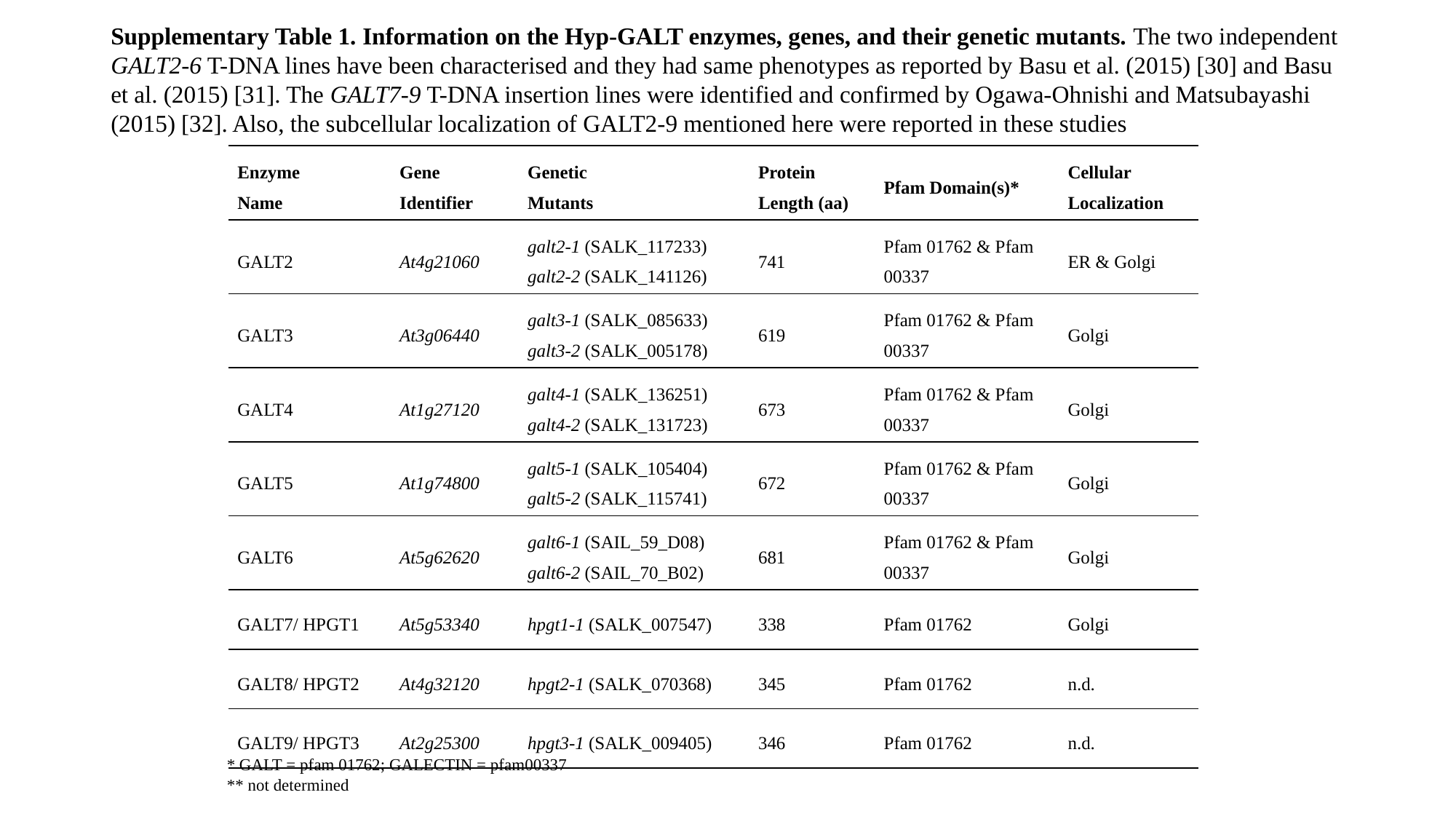

Supplementary Table 1. Information on the Hyp-GALT enzymes, genes, and their genetic mutants. The two independent GALT2-6 T-DNA lines have been characterised and they had same phenotypes as reported by Basu et al. (2015) [30] and Basu et al. (2015) [31]. The GALT7-9 T-DNA insertion lines were identified and confirmed by Ogawa-Ohnishi and Matsubayashi (2015) [32]. Also, the subcellular localization of GALT2-9 mentioned here were reported in these studies
| Enzyme Name | Gene Identifier | Genetic Mutants | Protein Length (aa) | Pfam Domain(s)\* | Cellular Localization |
| --- | --- | --- | --- | --- | --- |
| GALT2 | At4g21060 | galt2-1 (SALK\_117233) galt2-2 (SALK\_141126) | 741 | Pfam 01762 & Pfam 00337 | ER & Golgi |
| GALT3 | At3g06440 | galt3-1 (SALK\_085633) galt3-2 (SALK\_005178) | 619 | Pfam 01762 & Pfam 00337 | Golgi |
| GALT4 | At1g27120 | galt4-1 (SALK\_136251) galt4-2 (SALK\_131723) | 673 | Pfam 01762 & Pfam 00337 | Golgi |
| GALT5 | At1g74800 | galt5-1 (SALK\_105404) galt5-2 (SALK\_115741) | 672 | Pfam 01762 & Pfam 00337 | Golgi |
| GALT6 | At5g62620 | galt6-1 (SAIL\_59\_D08) galt6-2 (SAIL\_70\_B02) | 681 | Pfam 01762 & Pfam 00337 | Golgi |
| GALT7/ HPGT1 | At5g53340 | hpgt1-1 (SALK\_007547) | 338 | Pfam 01762 | Golgi |
| GALT8/ HPGT2 | At4g32120 | hpgt2-1 (SALK\_070368) | 345 | Pfam 01762 | n.d. |
| GALT9/ HPGT3 | At2g25300 | hpgt3-1 (SALK\_009405) | 346 | Pfam 01762 | n.d. |
* GALT = pfam 01762; GALECTIN = pfam00337
** not determined

## Slide 10
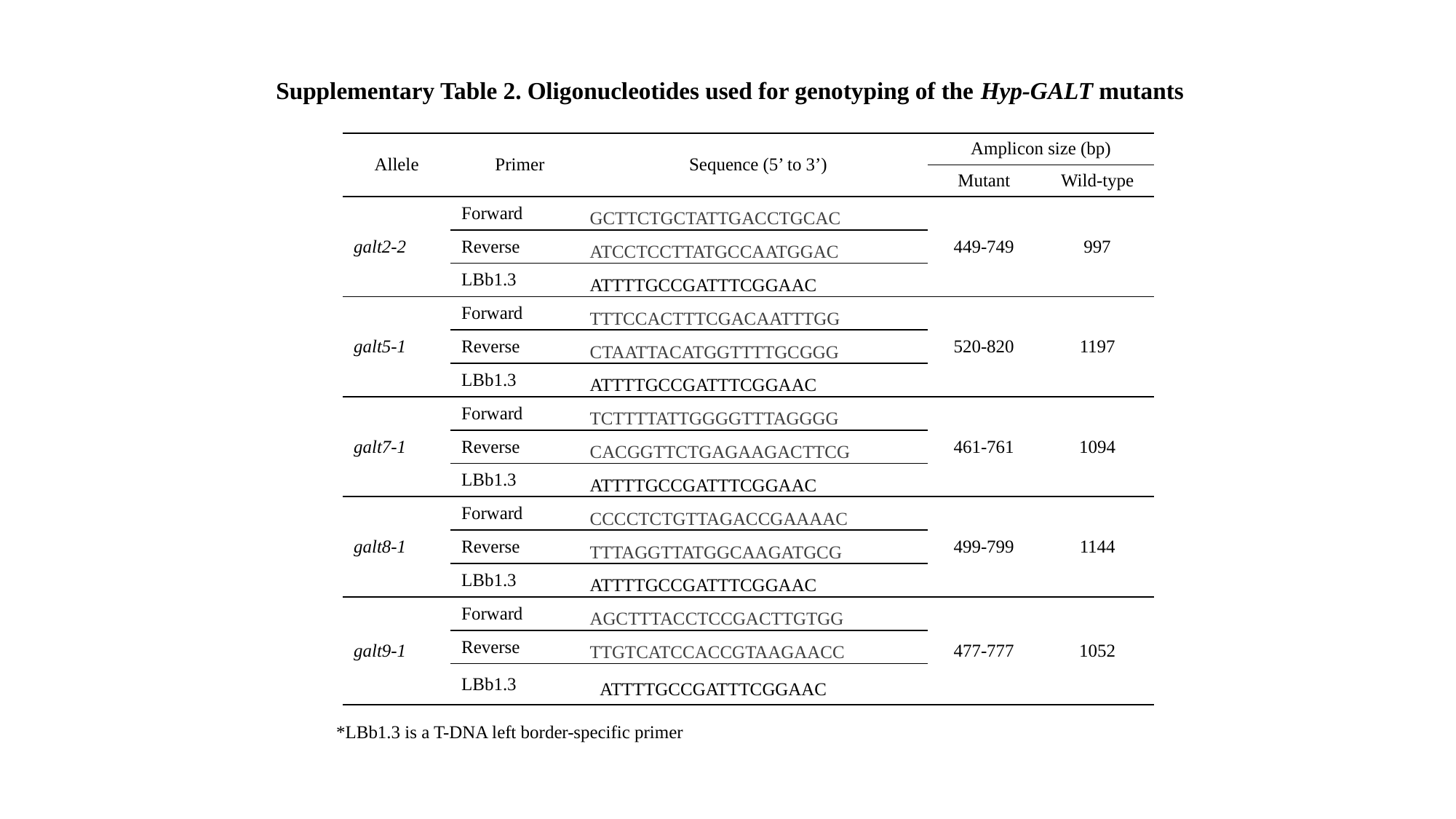

Supplementary Table 2. Oligonucleotides used for genotyping of the Hyp-GALT mutants
| Allele | Primer | Sequence (5’ to 3’) | Amplicon size (bp) | |
| --- | --- | --- | --- | --- |
| | | | Mutant | Wild-type |
| galt2-2 | Forward | GCTTCTGCTATTGACCTGCAC | 449-749 | 997 |
| | Reverse | ATCCTCCTTATGCCAATGGAC | | |
| | LBb1.3 | ATTTTGCCGATTTCGGAAC | | |
| galt5-1 | Forward | TTTCCACTTTCGACAATTTGG | 520-820 | 1197 |
| | Reverse | CTAATTACATGGTTTTGCGGG | | |
| | LBb1.3 | ATTTTGCCGATTTCGGAAC | | |
| galt7-1 | Forward | TCTTTTATTGGGGTTTAGGGG | 461-761 | 1094 |
| | Reverse | CACGGTTCTGAGAAGACTTCG | | |
| | LBb1.3 | ATTTTGCCGATTTCGGAAC | | |
| galt8-1 | Forward | CCCCTCTGTTAGACCGAAAAC | 499-799 | 1144 |
| | Reverse | TTTAGGTTATGGCAAGATGCG | | |
| | LBb1.3 | ATTTTGCCGATTTCGGAAC | | |
| galt9-1 | Forward | AGCTTTACCTCCGACTTGTGG | 477-777 | 1052 |
| | Reverse | TTGTCATCCACCGTAAGAACC | | |
| | LBb1.3 | ATTTTGCCGATTTCGGAAC | | |
 *LBb1.3 is a T-DNA left border-specific primer

## Slide 11
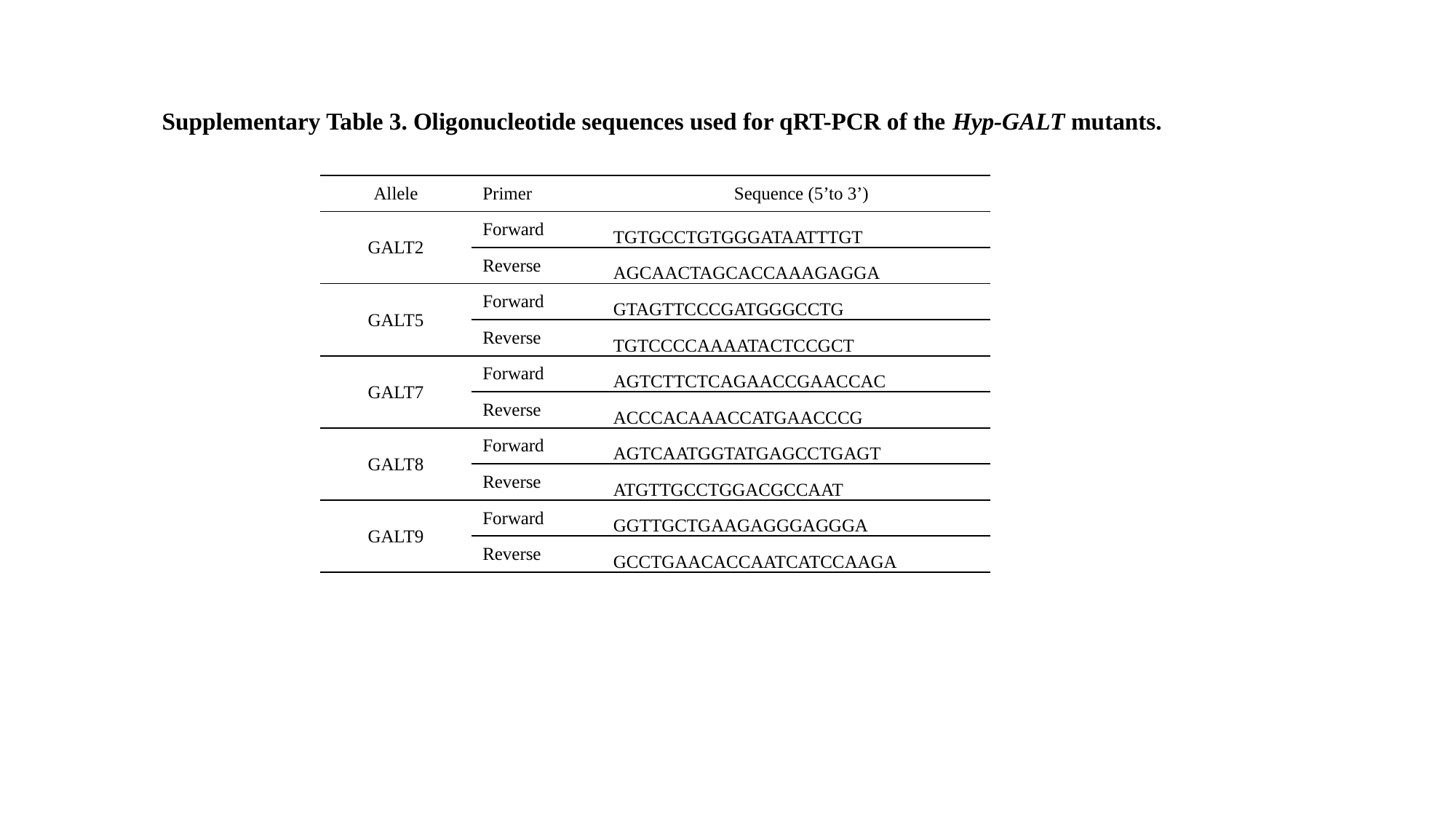

Supplementary Table 3. Oligonucleotide sequences used for qRT-PCR of the Hyp-GALT mutants.
| Allele | Primer | Sequence (5’to 3’) |
| --- | --- | --- |
| GALT2 | Forward | TGTGCCTGTGGGATAATTTGT |
| | Reverse | AGCAACTAGCACCAAAGAGGA |
| GALT5 | Forward | GTAGTTCCCGATGGGCCTG |
| | Reverse | TGTCCCCAAAATACTCCGCT |
| GALT7 | Forward | AGTCTTCTCAGAACCGAACCAC |
| | Reverse | ACCCACAAACCATGAACCCG |
| GALT8 | Forward | AGTCAATGGTATGAGCCTGAGT |
| | Reverse | ATGTTGCCTGGACGCCAAT |
| GALT9 | Forward | GGTTGCTGAAGAGGGAGGGA |
| | Reverse | GCCTGAACACCAATCATCCAAGA |

## Slide 12
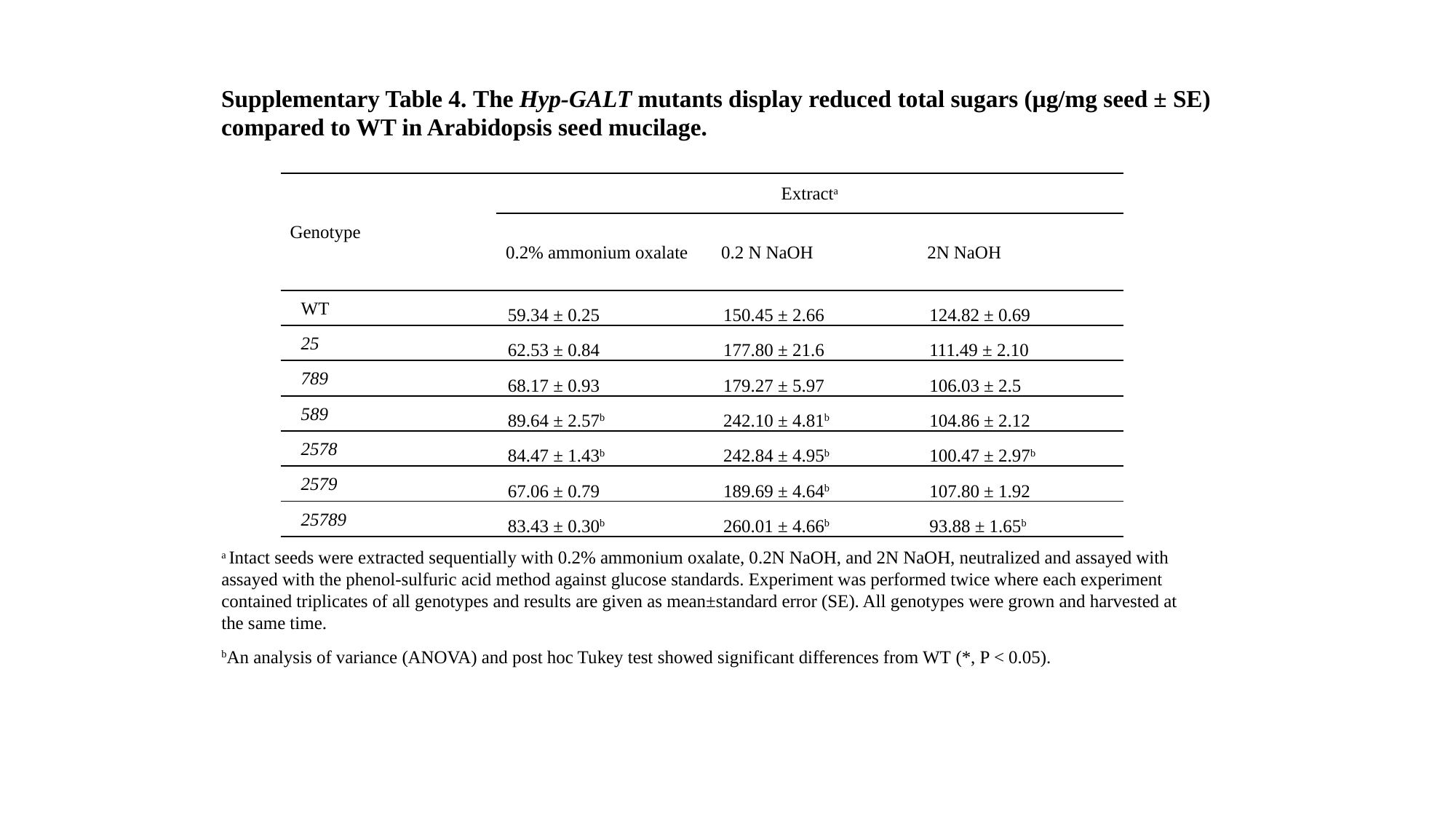

Supplementary Table 4. The Hyp-GALT mutants display reduced total sugars (µg/mg seed ± SE) compared to WT in Arabidopsis seed mucilage.
| Genotype | Extracta | | |
| --- | --- | --- | --- |
| | 0.2% ammonium oxalate | 0.2 N NaOH | 2N NaOH |
| WT | 59.34 ± 0.25 | 150.45 ± 2.66 | 124.82 ± 0.69 |
| 25 | 62.53 ± 0.84 | 177.80 ± 21.6 | 111.49 ± 2.10 |
| 789 | 68.17 ± 0.93 | 179.27 ± 5.97 | 106.03 ± 2.5 |
| 589 | 89.64 ± 2.57b | 242.10 ± 4.81b | 104.86 ± 2.12 |
| 2578 | 84.47 ± 1.43b | 242.84 ± 4.95b | 100.47 ± 2.97b |
| 2579 | 67.06 ± 0.79 | 189.69 ± 4.64b | 107.80 ± 1.92 |
| 25789 | 83.43 ± 0.30b | 260.01 ± 4.66b | 93.88 ± 1.65b |
a Intact seeds were extracted sequentially with 0.2% ammonium oxalate, 0.2N NaOH, and 2N NaOH, neutralized and assayed with assayed with the phenol-sulfuric acid method against glucose standards. Experiment was performed twice where each experiment contained triplicates of all genotypes and results are given as mean±standard error (SE). All genotypes were grown and harvested at the same time.
bAn analysis of variance (ANOVA) and post hoc Tukey test showed significant differences from WT (*, P < 0.05).
